# Double cross-linked graphene oxide hydrogel for promoting healing of diabetic ulcers

**DOI:** 10.3389/fchem.2024.1355646

**Published:** 2024-02-22

**Authors:** Wenxu Liu, Yunfang Yang, Meiying Li, Jingxin Mo

**Affiliations:** ^1^ Lab of Neurology, The Affiliated Hospital of Guilin Medical University, Guilin, China; ^2^ School of Pharmacy, Guilin Medical University, Guilin, China; ^3^ Health Management Centre, The Second Affiliated Hospital of Guilin Medical University, Guilin, China; ^4^ Clinical Research Center for Neurological Diseases of Guangxi Province, The Affiliated Hospital of Guilin Medical University, Guilin, China

**Keywords:** bioorthogonal click, graphene oxide, dual network hydrogel, diabetic ulcer damage, glutathione

## Abstract

This study explores the synthesis and characterization of a novel double cross-linked hydrogel composed of polyvinyl alcohol (PVA), sodium alginate (SA), graphene oxide (GO), and glutathione (GSH), henceforth referred to as PVA/SA/GO/GSH. This innovative hydrogel system incorporates two distinct types of cross-linking networks and is meticulously engineered to exhibit sensitivity to high glucose and/or reactive oxygen species (ROS) environments. A sequential approach was adopted in the hydrogel formation. The initial phase involved the absorption of GSH onto GO, which was subsequently functionalized with boric acid and polyethylene glycol derivatives via a bio-orthogonal click reaction. This stage constituted the formation of the first chemically cross-linked network. Subsequently, freeze-thaw cycles were utilized to induce a secondary cross-linking process involving PVA and SA, thereby forming the second physically cross-linked network. The resultant PVA/SA/GO/GSH hydrogel retained the advantageous hydrogel properties such as superior water retention capacity and elasticity, and additionally exhibited the ability to responsively release GSH under changes in glucose concentration and/or ROS levels. This feature finds particular relevance in the therapeutic management of diabetic ulcers. Preliminary *in vitro* evaluation affirmed the hydrogel’s biocompatibility and its potential to promote cell migration, inhibit apoptosis, and exhibit antibacterial properties. Further *in vivo* studies demonstrated that the PVA/SA/GO/GSH hydrogel could facilitate the healing of diabetic ulcer sites by mitigating oxidative stress and regulating glucose levels. Thus, the developed PVA/SA/GO/GSH hydrogel emerges as a promising candidate for diabetic ulcer treatment, owing to its specific bio-responsive traits and therapeutic efficacy.

## 1 Introduction

Type 2 diabetes (T2D) is a widespread metabolic disease that causes hyperglycemia (Maschalidi et al., 2022). A significant percentage of diabetics also develop bacterial infections in wounds such as diabetic ulcers (Sen, 2021), which become chronically infected and are a major clinical problem (Theocharidis et al., 2022). The primary causes of poor wound healing in diabetic ulcers are thought to be oxidative stress (Feng et al., 2022), compromised vasculogenesis (Rai et al., 2022), increased expression of pro-inflammatory factors (Rai et al., 2022), and bacterial infection (Armstrong et al., 2023).

In previous studies, glutathione (GSH) is a tripeptide that can act as an antioxidant to remove accumulated reactive oxygen species (ROS) (Xu et al., 2019) on the trauma surface and reduce the release of inflammatory factors (Boniakowski et al., 2019), thereby adjusting the trauma microenvironment. Graphene oxide (GO) has a structure similar to graphene (Girão et al., 2016) with a large number of hydroxyl groups and oxygen-containing functional groups on its carbon sheets (Mellado et al., 2018). In addition, GO has a large specific surface area, which facilitates physical drug loading and has potential antioxidant properties (Yin et al., 2022). We planned to adsorb GSH on GO, but the GO/GSH prepared in this way lacked the ability to deliver drugs continuously and release drugs responsively.

In recent years, researchers have designed and synthesized many biomaterials as carriers for the treatment of diabetic ulcers. Among them, hydrogels, as a three-dimensional network structured gel, can perfectly cover the irregular shape of wounds while having good water retention and exudate absorption capacity (Zhang B. et al., 2020). Sodium alginate is a linear polysaccharide derived mainly from brown algae. It is widely used in biomedical applications due to its biocompatibility and gel-forming ability (Thakur et al., 2018). When polyvinyl alcohol (PVA) is introduced, the water-soluble mixture of PVA and SA has good biocompatibility (Kamoun et al., 2017). PVA-based hydrogels can generate cross-linked networks by physical freeze-thawing without the use of toxic cross-linking agents (Niu et al., 2021). The combination of PVA and SA to form hydrogels using freeze-thaw crosslinking methods has been previously reported (Zhang J. et al., 2020). However, simple mixing of GO/GSH into PVA/SA-based hydrogels allows for continuous drug delivery but does not allow for responsive drug release based on changes in ROS and glucose concentrations at the trauma site.

Bioorthogonal reactions are chemical reactions that take place in living organisms and that do not interfere with the natural biochemical processes in the organism (Ngai et al., 2022). Also, click chemistry is a method that uses simple and versatile small molecule chemical reactions to bind to biological molecules without producing by-products. Boronic acid derivatives can form reversible cyclic boron ester bonds by binding well to substances with an adjacent dihydroxy group (Zhao et al., 2013). Cycloboronate bonds have high glucose and/or ROS sensitivity (Zhao et al., 2013; Stubelius et al., 2019). In the presence of high glucose and/or high ROS, Cycloboronate bond breakage can occur, resulting in the release of glutathione. Polyethylene glycol (NH_2_-PEG-COOH) has been used to make drug carriers, which when coupled to the parent, increase the hydrodynamic radius of the parent, thereby increasing biocompatibility (Monge et al., 2021). The above synthetic pathway reactions require mild conditions and are highly specific, satisfying the basic concepts of bioorthogonal click chemistry (Ngai et al., 2022).

**SCHEME 1:**
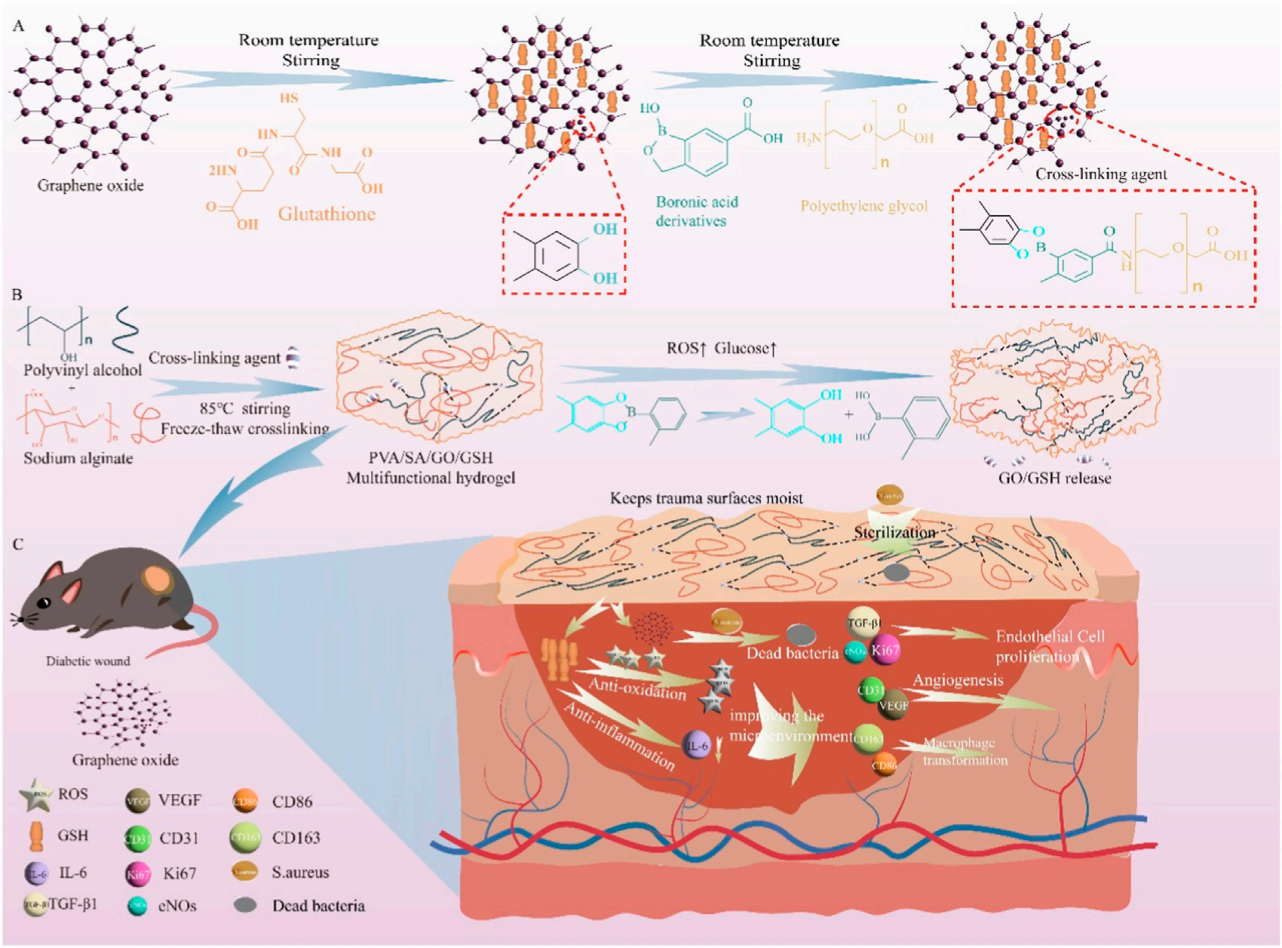
This study utilized a bioorthogonal click modified GSH -loaded GO cross-linked hydrogel for the specific treatment of ulcer damage in diabetic mice by reducing oxidative stress and inflammation. The scheme includes **(A)** the synthesis of the cross-linking agent, **(B)** the preparation and breakage of the PVA/SA/GO/GSH hydrogel under high glucose and/or high ROS, and **(C)** the treatment of diabetic ulcer using the hydrogel.

Therefore, in this study, this cross-linking agent is a GO physically adsorbed GSH, and then the adjacent dihydroxy groups on GO and boronic acid derivatives reversibly form cyclic boronic esters to form the first layer of chemical cross-linked network. The agent is directly copolymerized with PVA and SA, and then frozen to form a second physical cross-linked network, forming the target smart double network PVA/SA/GO/GSH hydrogel. Subsequently, we systematically evaluated the mechanical properties, release response properties of this dual network hydrogel, explored the *in vitro* biocompatibility and cell migration ability of PVA/SA/GO/GSH hydrogel, and investigated the *in vitro* ROS scavenging efficiency and antibacterial ability of the hydrogel. Finally, we evaluated the ROS clearance efficiency, wound healing ability and related therapeutic mechanisms of hydrogels *in vivo* (Scheme 1).

## 2 Materials and methods

### 2.1 Materials

The chemical reagents used in the study were purchased from various sources. 1-hydroxy-1,3-diamine-2,1-benzoxazole-6-carboxylic acid was bought from Yuanye Biotechnology Co., Ltd. (Shanghai, China), GO from Jiangsu XFNANO Materials Tech. Co., Ltd. (Nanjing, China), and NH2-PEG-COOH from Ponsure Biotechnology Co., Ltd. (Shanghai, China). All other chemical reagents were obtained from Aladdin Reagents Co., Ltd. (Shanghai, China). Kits from Beyotime Biotechnology Co., Ltd. (Shanghai, China) were used except for the CCK-8 assay kit, which was purchased from MedChemExpress (America).

### 2.2 Cells

The NCTC clone 929 (L-929) mouse fibroblast cell line was obtained from the CTCC of Science (Shanghai, China). The cells were cultured in modified eagle medium (MEM) from Gibco, supplemented with 10% fetal bovine serum from Gibco and 100 U/mL penicillin and 100 μg/mL streptomycin from Gibco.

### 2.3 Animals

Male C57BL/6 mice, aged 10 weeks, were obtained from Hunan SJA Laboratory Animal Co., Ltd (Changsha, China). The animal experimental protocol was approved by the Animal Ethics Committee of Guilin Medical University (ethics number GLMC-202105006). The mice were housed in SPF-level animal rooms with a 12-h light/dark cycle, an ambient temperature of 26°C, and a humidity of 50%. The mice were given access to sterile food and water.

### 2.4 Preparation of cross-linking agent

The crosslinker was synthesized according to the previous literature with some modifications (Akgun and Hall, 2016; Csga et al., 2019). The specific experimental steps are as follows.

First, GO (0.4 mg, 2 mg/mL) and GSH (Mw 307.33, 4 µM) were mixed uniformly in water and stirred at room temperature for 24 h to allow GSH to be adsorbed on GO; then 1-hydroxy-1,3-dihydrobenzo[c] (Sen, 2021; Maschalidi et al., 2022)oxaborolane-6-carboxylic acid (Mw 177.95, 1.5 mg, 8.4 µM) was dissolved in DMF (20 mL) and triethylamine (Mw 101.19, 1.7 mL, 16.8 µM) and NH_2_-PEG-COOH (Mw 10,000, 40 mg, 4 µM) were added at room temperature. After mixing and stirring for 10 min, GO/GSH (0.4 mg, 2 mg/mL) or GO (0.4 mg, 2 mg/mL) was added separately and the mixture was kept stirring for 16 h at room temperature, centrifuged at 12,000 rpm for 30 min at room temperature, the supernatant was discarded and the precipitate was taken and dispersed in 15 mL of physiological saline. The one with GO added is called cross-linker A and the one with GO/GSH added is called cross-linker B. The amounts of GO and GSH are shown in Supplementary Figure S1. To obtain ^1^H NMR spectra, the NMR-AVANCE NEO 500 MHz apparatus was used. GSH, GO, and the Cross-linking Agent were dissolved in D_2_O for the analysis. Fourier transform infrared spectroscopy (FTIR, Thermo 6700, America) was used to conduct hydrogel chemical structural analysis, with a range of 400–4,000 cm^−1^.

### 2.5 Preparation and characterization of PVA/SA/GO/GSH hydrogel for the treatment of diabetic ulcer damage

To prepare the hydrogels, 2 g of PVA was weighed and dissolved in 20 mL of cross-linking agent A, while 2 g of PVA and SA (ratio 9:1) were dissolved separately in 20 mL of the same cross-linking agent. The solutions were heated in a water bath at 85°C and stirred to form a 10% clarified solution. The solutions were then frozen overnight at −80°C, and upon restoration to room temperature, the PVA/GO hydrogel and PVA/SA/GO hydrogel were formed.

To obtain the PVA/SA/GO/GSH hydrogel, 2 g of PVA and SA (ratio 9:1) were dissolved in 20 mL of cross-linking agent B, and the same preparation method as above was followed.

Various evaluations, such as hydrogel tensile analysis, gel formation time analysis, scanning electron microscopy (SEM HITACHI SU8100) analysis, water retention ratio, swelling ratio, and electrical conductivity characteristics, were performed. The experimental details are presented in the Experimental Details section of the Supplementary Material.

### 2.6 Glutathione release from PVA/SA/GO/GSH hydrogel under different circumstances

The *in vitro* release properties of glutathione in the hydrogels were studied using PBS with varying concentrations of glucose or H_2_O_2_ at physiological temperature (37°C) and pH (7.4). The experimental details are provided in the Supplementary Material.

### 2.7 Cytocompatibility evaluation of PVA/SA/GO/GSH hydrogel

The cytocompatibility of the PVA/SA/GO/GSH hydrogel was evaluated through hemolytic activity tests on red blood cells and L929 cell viability tests. Additionally, cell scratch and transwell experiments were performed to assess cell growth and migration after treatment with the hydrogel. The experimental details can be found in the Supplementary Material.

### 2.8 *In vitro* apoptosis assessment

To assess apoptosis, cells were initially cultured in MEM supplemented with 1 mM H_2_O_2_ for 1 hour to simulate oxidative stress conditions relevant to diabetic environments. Following this incubation, the medium was discarded and the cells were exposed to MEM containing various treatments. After 2 days, these cells were harvested, washed, and analyzed using an Annexin V/fluorescein isothiocyanate (FITC) kit from BD Biosciences. After staining cells with FITC Annexin V for 15 min while protected from light, they were stained with Propidium Iodide Staining Solution. The apoptosis levels were evaluated through flow cytometry using an Agilent Technologies instrument.

### 2.9 Bacterial inhibition and ROS scavenging abilities of PVA/SA/GO/GSH hydrogel

The bacterial inhibition ability of the PVA/SA/GO/GSH hydrogel was evaluated through the formation of inhibition circles, while the *in vitro* ROS scavenging ability of the hydrogel was assessed using fluorescence intensity of ROS probes. The experimental details are presented in the Supplementary Material.

### 2.10 Evaluation of the effect of PVA/SA/GO/GSH hydrogel on diabetic mouse model for wound healing assessment

A diabetic mouse model was established to evaluate the effect of PVA/SA/GO/GSH hydrogel on wound healing. Male C57BL/6 mice were randomly divided into five groups, including Control, GSH, PVA/GO hydrogel, PVA/SA/GO hydrogel, and PVA/SA/GO/GSH hydrogel. The mice were treated with either PBS, GSH, or the respective hydrogel daily until the end of the experiment. Wound healing rates were evaluated on days 0, 3, 7, and 12 after wound formation, and the wound healing ratio was calculated as follows:

$$\text{The rate of wound healing}\left(\%\right)=\frac{S_{0}‐S_{t}}{S_{0}}\times 100\%$$

Wherein S_0_ denotes wound size on day 0 and S_t_ denotes wound size at specified time points.

The wound tissue was collected on days 3, 7, and 12, fixed in 4% paraformaldehyde, embedded, and sectioned for histological analysis. Hematoxylin-eosin (H&E) and Masson’s trichrome staining were used to assess wound healing. Immunohistochemical staining for Ki67, VEGF, CD31, CD86, and CD163 was performed to examine inflammation, angiogenesis, and macrophage transformation at the wound site. Further details on the experimental procedures can be found in the Supplementary Material.

### 2.11 *In vivo* assessment of the antioxidant and antiglucose effects of PVA/SA/GO/GSH hydrogels

We evaluated the antioxidant effects of PVA/SA/GO/GSH hydrogels during wound healing by measuring the levels of MDA, SOD, ROS, NO and glucose in skin tissues on days 3, 7, and 12 after grinding and homogenizing them. We also monitored the ROS-mediated inflammatory activity at the wound site using an *In vivo* Imaging system to gain insight into the antioxidant role of PVA/SA/GO/GSH hydrogels. To do this, we used the ROS probe DCFH-DA (100 µM) and recorded fluorescence images with mean fluorescence intensity values. The operation steps are presented in the Experimental Details in the Supplementary Material.

### 2.12 Evaluation of TNF-α and IL-6 protein expression levels in hydrogel-treated skin tissues

Skin tissues from each group were lysed using RIPA buffer, and the concentration of the samples was quantified by BCA protein. Equivalent volumes of protein were loaded onto 15% sodium dodecyl sulfate-polyacrylamide gel electrophoresis and transferred to poly (vinylidene fluoride) films. The membranes were incubated with anti-TNF-α and anti-IL-6 antibodies and exposed with chemiluminescent substrate. The expression levels of TNF-α and IL-6 proteins were evaluated. The measurement of IL-6, TGF-β_1_, and VEGF in tissues using ELISA kits was also performed.

### 2.13 *In vivo* biosafety assessment

We conducted a skin irritation test to evaluate the potential irritation response of the hydrogel to the organism. The hydrogel was applied to the left side of the de-haired mouse’s back, while the right side was used as the Control group with saline-absorbed gauze. Skin response was observed and recorded after 24, 48, and 72 h. Skin irritation response grading criteria presented in Supplementary Table S1 were used to assess the response.

To evaluate the potential irritation of the hydrogel on internal organs, mice were perfused on day 12 after trauma, and HE staining was performed on the heart, liver, spleen, lungs, kidneys, stomach, intestines, and brain to observe the structures.

### 2.14 Statistical analysis

Unless otherwise indicated, all data are presented as mean ± standard deviation (SD). Statistical significance was evaluated by one-way ANOVA with Tukey’s multiple comparison test. A *p*-value less than 0.05 was considered statistically significant.

## 3 Results

### 3.1 Preparation, characterization, and mechanical properties of hydrogels

**FIGURE 1:**
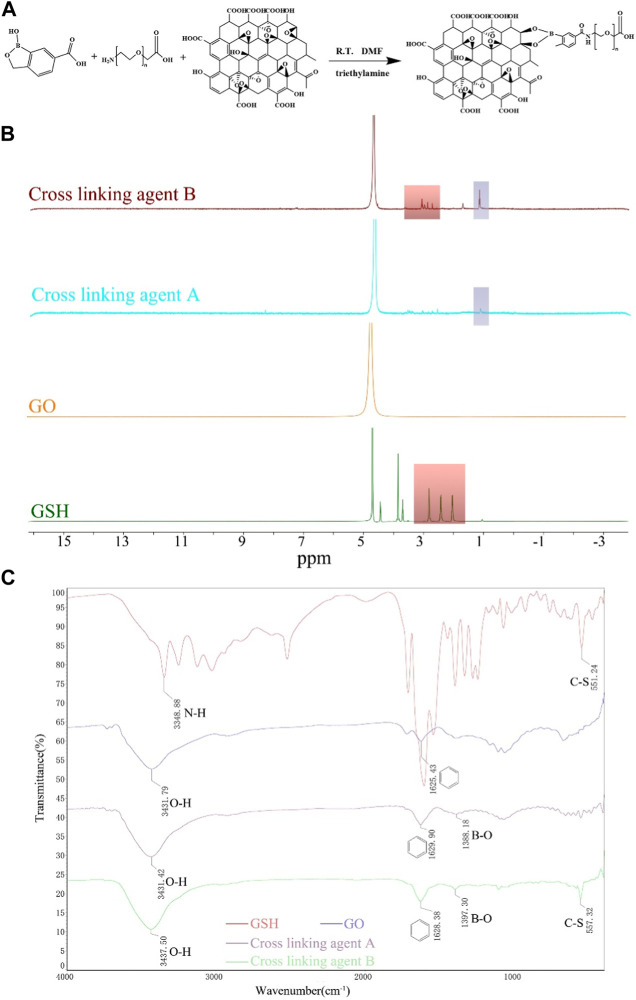
Illustration of the polymer’s synthetic pathway, complemented by its characterization through ^1^H-NMR and FTIR spectra. **(A)** Synthetic pathway illustrating the formation of the polymer using click bioorthogonal coupling. **(B)** ^1^H-NMR spectra of GO, GSH, Cross-linking agent A, and Cross-linking agent **(B) (C)** FTIR spectra of GSH, GO, Cross-linking agent **(A)**, and Cross-linking agent **(B)**.

The hydrogel synthesized in our study exhibits several notable characteristics. Firstly, it possesses both anti-inflammatory and antioxidant properties. Notably, the cross-linking agent used for the hydrogel’s formation was derived through a singular “one-pot copolymerization” approach, utilizing click bioorthogonal coupling, as depicted in Figure 1A (Zhao et al., 2013).

The successful synthesis of the cross-linking agent was confirmed by ^1^H-NMR spectroscopy and FTIR analysis. The ^1^H NMR spectrum of cross-linking Agent B showed a peak at ∼3.70 ppm, indicating the successful adsorption of GSH. And the resonance peak signal of the boronic acid derivatives can be seen at ∼1.20 ppm of both crosslinkers A/B (Figure 1B).

The FTIR spectrum (Figure 1C) revealed that in addition to the appearance of the benzene ring backbone near 1,600 cm^−1^ and O-H vibrations near 3,500 cm^−1^ in cross-linking agent A/B, a new peak was detected at 1,380–1,400 cm^−1^, representing the stretching vibration of B-O. Moreover, the C-S stretching vibration of GSH appeared at 557.32 cm^-1^ in cross-linking agent B.

These results confirm the successful grafting of boronic acid derivatives onto GO and the successful adsorption of GSH into cross-linking agent B, indicating the successful synthesis of the cross-linking agent.

### 3.2 Characterization, and mechanical properties of hydrogels

The hydrogel, synthesized via the described method, demonstrated inherent tissue-adhesive properties, ensuring facile application and residue-free removal from skin tissues as evidenced in Supplementary Figure S2A. The material also showcased appreciable stretchability, depicted in Supplementary Figure S2B. Notably, the combined PVA/SA solution with the cross-linking agent remained in a sol state at 85°C, but underwent a sol-gel transition within 2 h, as delineated in Supplementary Figure S2C. Subsequent to a freeze-crosslinking process, a complete transition to a gel state was achieved.

**FIGURE 2:**
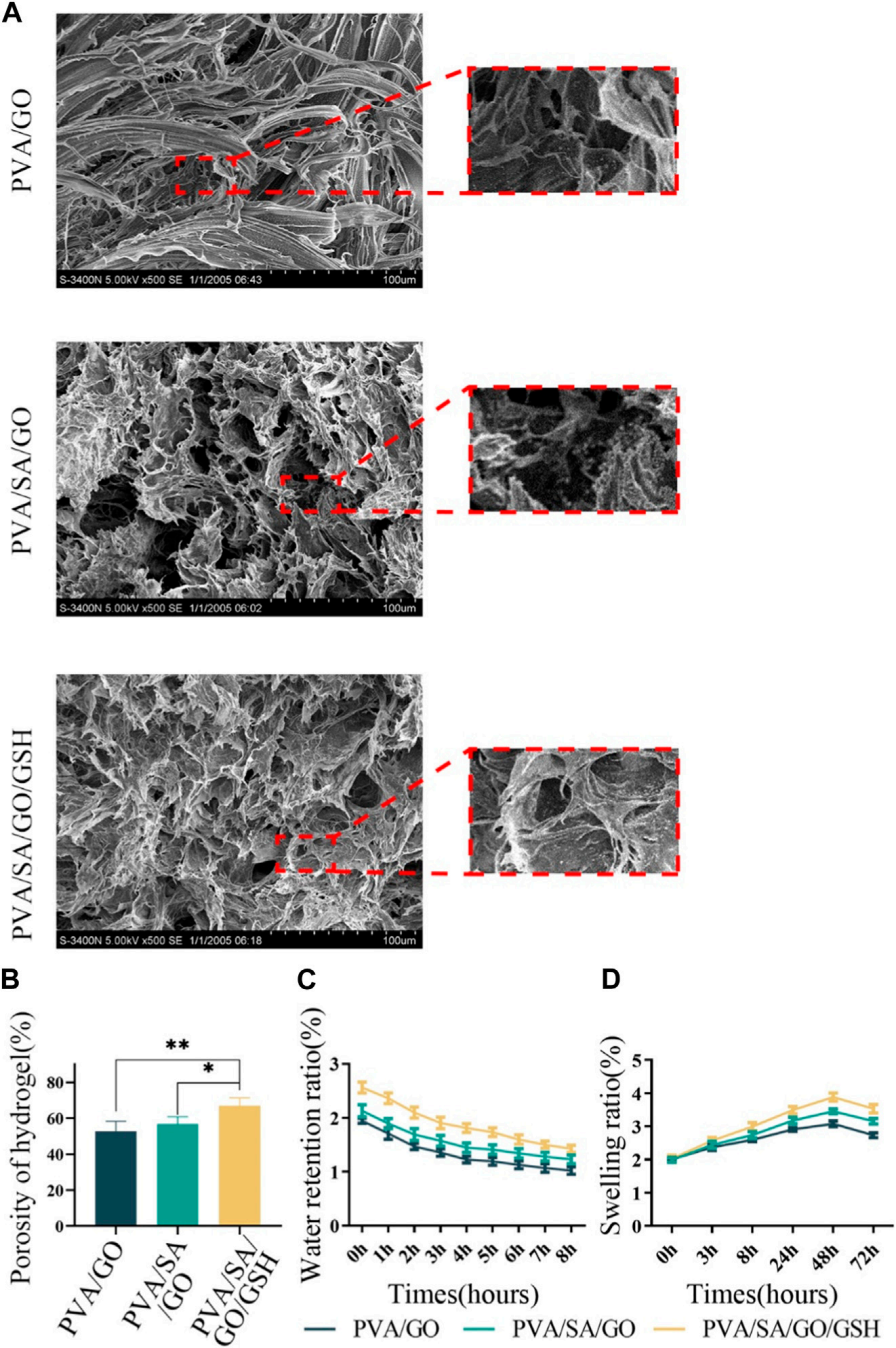
Comprehensive physical assessments of the hydrogels. **(A)** Scanning electron microscopy (SEM) images illustrating hydrogel microstructure. **(B)** Quantitative analysis of hydrogel porosity. **(C)** Analysis of the hydrogels’ water retention capacities. **(D)** Analysis of the hydrogels’ swelling behaviors. Data are reported as means ± standard deviation and **p ≤ 0.01, *p ≤ 0.05.

The hydrogel samples were analyzed using SEM, revealing that they all possessed sparse and porous structures, which provided them with adequate permeability (Figure 2A). The PVA/GO hydrogel had a porosity of 52.64% and an average pore size of 71.75 µm, while the PVA/SA/GO composite hydrogel had a porosity of 56.85% and an average pore size of 65.61 µm. On the other hand, the PVA/SA/GO/GSH composite hydrogel exhibited a significantly smaller average pore size of 59.00 µm and higher porosity of 66.97%. The increase in cross-linked components led to a higher cross-linked density, resulting in a smaller pore size, complex porous structure, and increased porosity (Figure 2B).

To confirm the binding between the hydrogel backbone and the crosslinker, we conducted FTIR analysis (Supplementary Figure S3). In all three types of hydrogels, we observed prominent vibrations of the aromatic ring backbone and stretching vibrations of B-O, which were attributed to GO and boronic acid derivatives in the crosslinking agent. We also detected C-S stretching vibrations in the PVA/SA/GO/GSH hydrogel, providing evidence for the presence of GSH in this hydrogel.

We assessed the water retention, swelling, and electrical conductivity properties of the hydrogels to evaluate their potential for promoting wound healing (Liang et al., 2019; Zhao et al., 2021). The PVA/SA/GO/GSH composite hydrogel exhibited superior water retention (Figure 2C) and swelling properties (Figure 2D) compared to the other hydrogel components. The intricate process of hydrogel synthesis, albeit complex, substantially enhances the overall performance of the resultant hydrogel as a therapeutic wound dressing. While individual components of the hydrogel might not exhibit significant differential properties, their synergistic interaction through a layered cross-linking network amplifies the hydrogel’s effectiveness.

Interestingly, the physisorption of GSH onto the hydrogel network does not compromise the inherent conductive properties of the hydrogel. Empirical evaluation through electrical conductivity analyses reaffirmed this observation, asserting that the presence of GSH does not alter the electrical characteristics of the hydrogel (Supplementary Figure S4).

Cumulatively, these results underline the potential of the developed hydrogel as a promising candidate for wound healing applications. Its multi-layered architecture, responsive behavior, and unaffected conductivity collectively contribute to its therapeutic potential, bolstering its candidacy for advanced wound care solutions, particularly in diabetic ulcer management.

### 3.3 Responsive drug release from hydrogel

**FIGURE 3:**
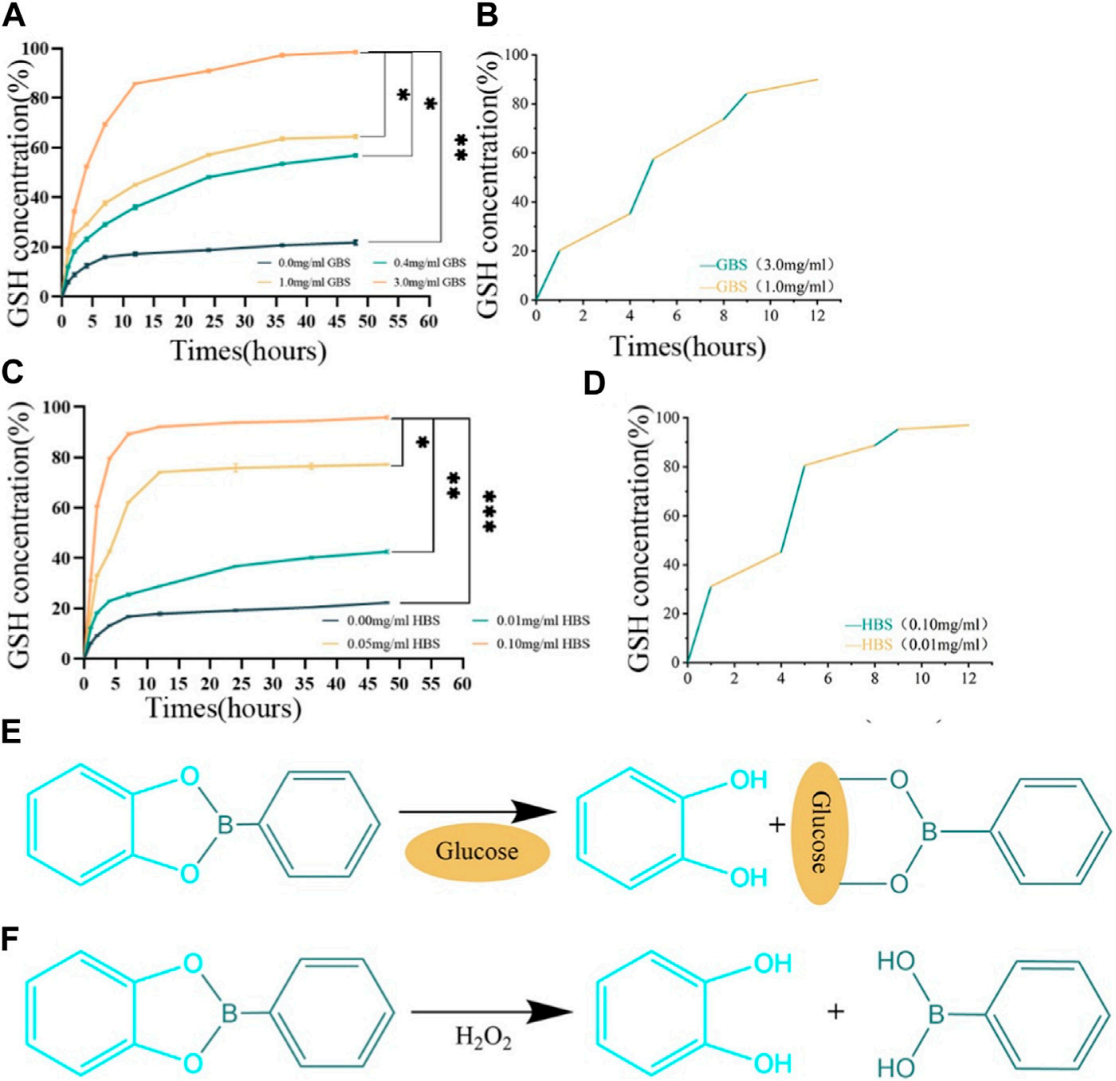
Responsive drug release properties of the hydrogels. **(A)** Release curves of PVA/SA/GO/GSH hydrogels in GBS with different concentration gradients. **(B)** Cyclic release curves of PVA/SA/GO/GSH hydrogels in GBS with high or low concentration gradients. **(C)** Release curves of PVA/SA/GO/GSH hydrogels in HBS with different concentration gradients. **(D)** Cyclic release curves of PVA/SA/GO/GSH hydrogels in HBS with high or low concentration gradients. **(E)** The reaction equation between cyclic boron ester bond and glucose. **(F)** The reaction equation between cyclic boron ester bond and H_2_O_2_. Data are reported as means ± standard deviation and ***p ≤ 0.001, **p ≤ 0.01, *p ≤ 0.05.

We systematically analyzed the release kinetics of GSH from the PVA/SA/GO/GSH hydrogels in high glucose and/or elevated H_2_O_2_ environments. In Figure 3A, GSH release was distinctly enhanced in glucose buffer saline (GBS) relative to phosphate buffer saline (PBS). Remarkably, a GBS concentration of 3.0 mg/mL catalyzed the maximum GSH liberation, reaching an impressive 98% of the total encapsulated volume within 48 h. Delving into the release kinetics further, Figure 3B highlights the pronounced rate discrepancy: GSH release in 3.0 mg/mL GBS was precipitous within the initial hour, in stark contrast to the more protracted 3-h release observed in 0.4 mg/mL GBS.

Turning our attention to the influence of H_2_O_2_, Figure 3C indicates that higher concentrations of H_2_O_2_ buffer saline (HBS) expedited GSH release. Notably, nearly 97% of the total GSH was liberated within 48 h in this elevated H_2_O_2_ environment. Figure 3D accentuates this point by underscoring the brisk release kinetics in 0.1 mg/mL HBS, surpassing all other studied groups.

In Figures 3E, F, the depicted reaction equations highlight the hydrogel’s intelligent design, which allows the cyclic boron ester bond to respond to physiological signals. Figure 3E reveals how the bond interacts with glucose, enabling a glucose-mediated release of glutathione, whereas Figure 3F demonstrates the bond’s sensitivity to oxidative stress, specifically to H_2_O_2_, triggering the release of glutathione in response to increased ROS levels. This dual-responsive mechanism underscores the hydrogel’s potential for targeted therapeutic delivery in the complex wound environment of diabetic ulcers.

The observed rapid release dynamics can be rationalized by the cyclic boron ester bond’s presence. This bond, orchestrated by neighboring dihydroxyl groups on boronic acid derivatives and GO, facilitates the accelerated GSH release in contexts of heightened glucose or H_2_O_2_ concentrations.

### 3.4 Hydrogel-mediated biocompatibility and inhibition of apoptosis

**FIGURE 4:**
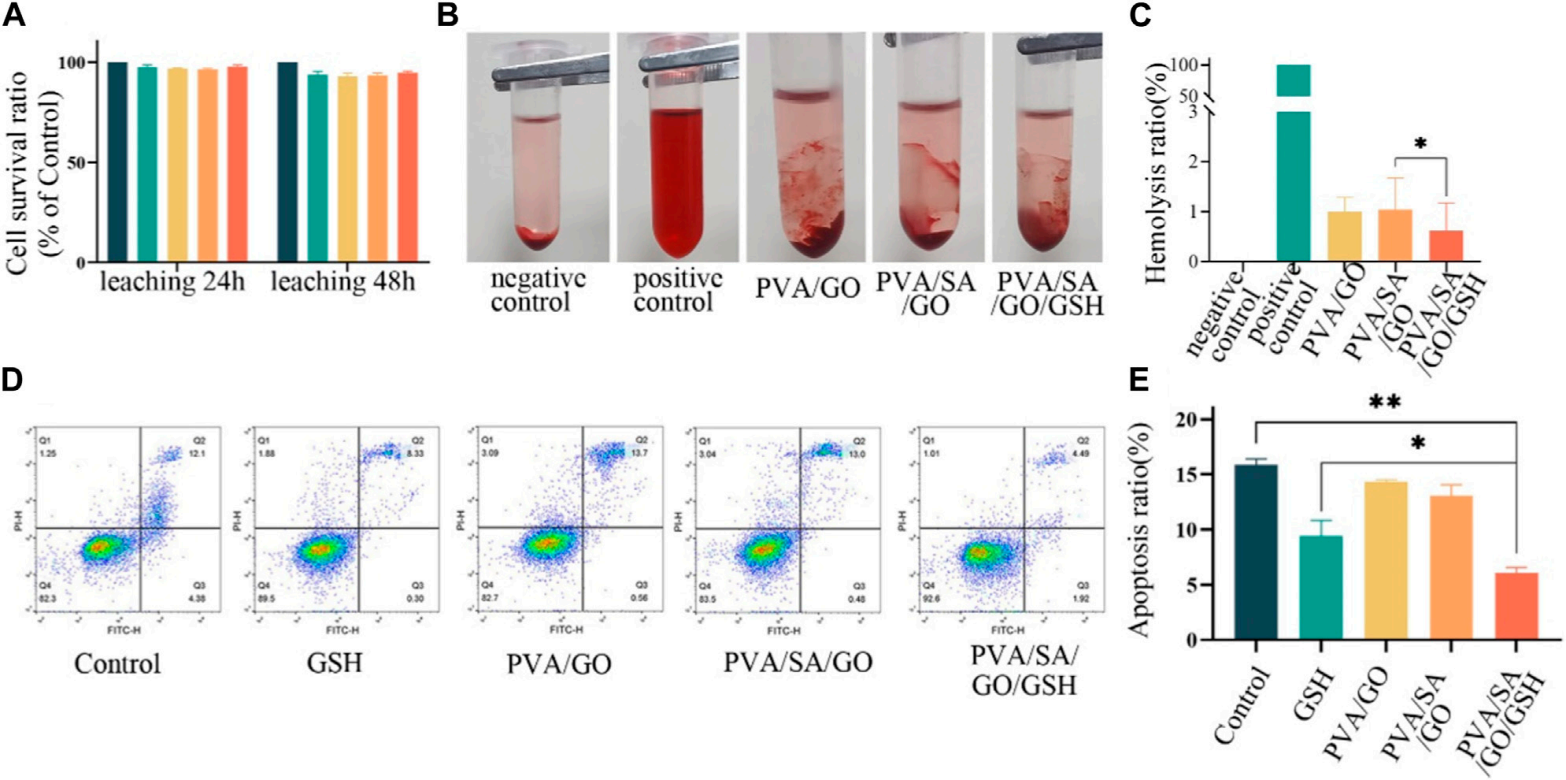
*In vitro* biocompatibility anti-apoptotic effect of various hydrogel treatments. **(A)** The viability of L929 cells following 24 and 48-h treatments with the hydrogels. **(B)** Photographs of different hydrogel hemolysis assays and **(C)** Histogram of hemolysis ratios. **(D)** Flow cytometry assessment of apoptosis in L929 cells after different treatments. The control group is without any treatment. and **(E)** Histogram of apoptosis ratios. Data are presented as means ± standard deviation. Statistically significant differences are denoted by asterisks, with *p ≤ 0.05, **p ≤ 0.01.

Cell viability was evaluated after 24 and 48 h of culture in hydrogel leachate. The survival rates for all experimental and control groups exceeded 95% with no statistically significant difference observed (Figure 4A).

The hemolysis rate of hydrogel materials is an important safety standard, with a rate less than 5% being acceptable (Chetouani et al., 2022). To evaluate the hemocompatibility of the various hydrogel compositions, we determined their hemolytic rates. The resulting values were 1.00%, 1.03%, and 0.62% for PVA/SA, PVA/SA/GO, and PVA/SA/GO/GSH hydrogels respectively. These results, significantly below the defined safety threshold, are indicative of the hydrogels’ favorable hemocompatibility. Notably, the incorporation of GSH into the hydrogel formulation resulted in a marked reduction in the hemolysis rate in comparison to the other groups (Figures 4B, C).

The potential anti-apoptotic effect of the PVA/SA/GO/GSH hydrogel was also examined using flow cytometry to detect apoptotic cells. A comparison with the control group revealed that the GSH and PVA/SA/GO/GSH hydrogel groups exhibited a decline in the percentage of apoptotic cells from 16.48% to 8.63% and 6.41% respectively (Figures 4D, E). These findings underscore the beneficial attributes of the PVA/SA/GO/GSH hydrogel, namely, its hemocompatibility and anti-apoptotic effect, which render it a promising candidate for therapeutic wound dressing applications.

### 3.5 Hydrogel-mediated cell migration, ROS scavenging and *in vitro* antimicrobial capacity

**FIGURE 5:**
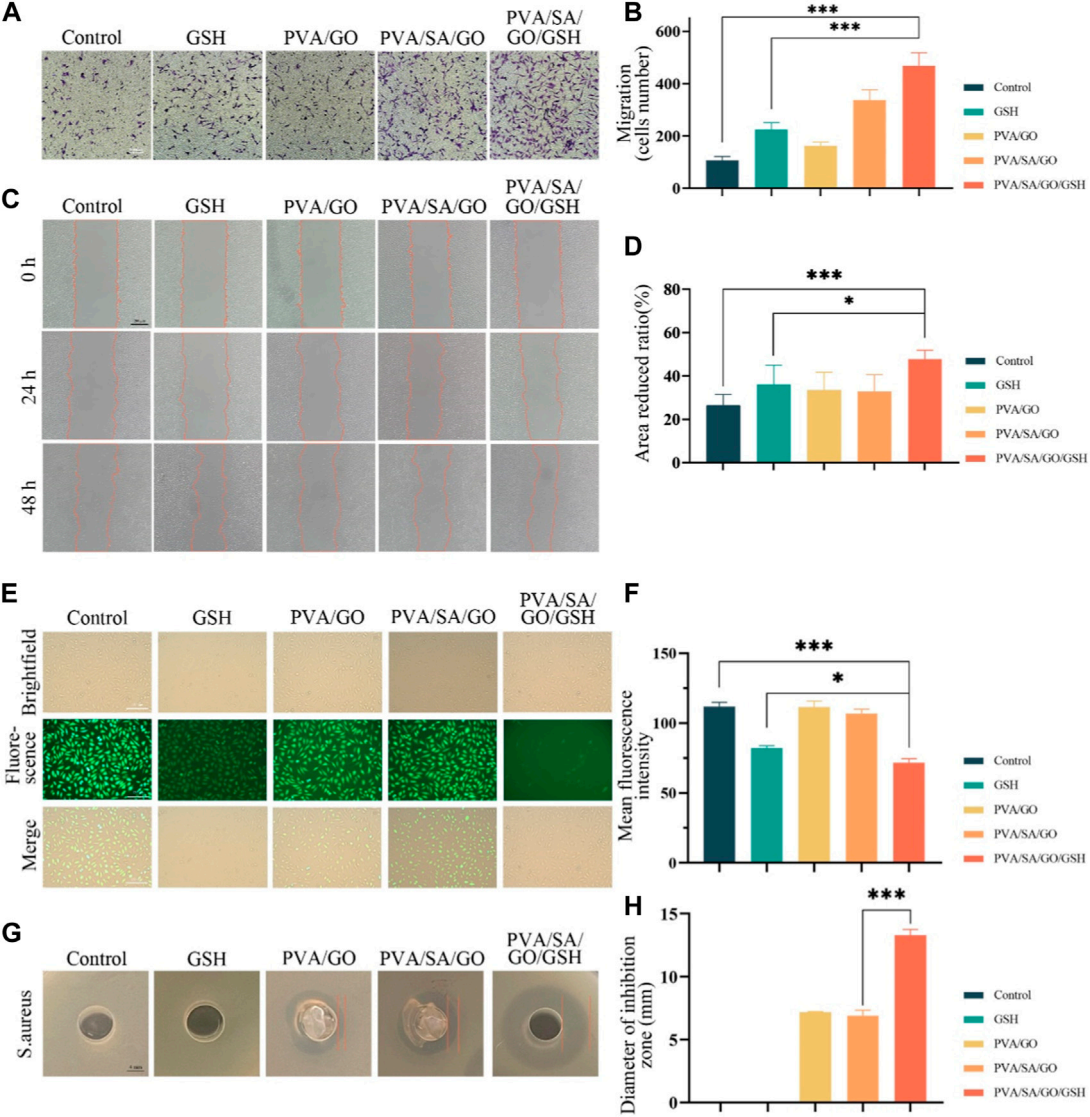
Evaluation of the hydrogel’s efficacy in cell migration, antioxidative activity, and bacterial inhibition. **(A)** Depicts cell migration assessed via the transwell method. **(B)** Quantitative analysis of cell migration from the transwell assay. **(C)** Visual representation of cell migration using the scratch assay. **(D)** Statistical interpretation of the scratch assay results. **(E)** Fluorescence microscopy images of ROS-induced L929 cells post-incubation with DCFH-DA. **(F)** Corresponding quantitative analysis of fluorescence intensity, indicative of antioxidative capability. **(G)** The zones of bacterial inhibition (against *S. aureus*) resulting from various hydrogel treatments. **(H)** A quantitative measure of the observed bacterial inhibition diameters. Data are presented as means ± standard deviation. Statistically significant differences are denoted by asterisks, with *p ≤ 0.05, ***p ≤ 0.001.

Cell migration, a critical determinant in wound healing, was assessed using transwell and scratch assays. Relative to the control group, each hydrogel-treated group demonstrated a significant enhancement in cell migration. Interestingly, the PVA/SA/GO/GSH hydrogel was superior to the GSH group in promoting cell migration (Figures 5A, B). This observation was corroborated by the scratch assay, which showed that hydrogel-treated cells migrated at a faster rate than the control group. Remarkably, the PVA/SA/GO/GSH hydrogel was found to have the greatest effect on cell migration, with a wound closure rate nearing 60% at 48 hours. This was significantly higher than the GSH and control groups (Figures 5C, D).

The ability of the hydrogels to scavenge reactive oxygen species (ROS) was evaluated using ROS-up agent-treated L929 fibroblasts, which were subsequently incubated with the hydrogels. The ROS concentration was determined by using 2′,7′-dichlorodihydrofluorescein diacetate (DCFH-DA) as a probe, which upon oxidation, produces fluorescent dichlorofluorescein (DCF). Figure 5E demonstrates a decrease in fluorescence intensity for all hydrogel-treated L929 cells compared to the control group, suggesting a reduction in ROS concentration. Notably, the most significant decrease in fluorescence was observed in cells treated with the PVA/SA/GO/GSH hydrogel (Figure 5F), indicating the superior ROS scavenging capacity of this hydrogel amongst those tested.

Furthermore, bacterial infections can pose significant impediments to wound healing, particularly in chronic wounds. Consequently, the incorporation of antimicrobial properties into wound dressings may offer substantial clinical advantages (Tyeb et al., 2020). The antimicrobial potential of the hydrogels was evaluated in this study, with a particular focus on their efficacy against *Staphylococcus aureus* (*S. aureus*). An assessment of the diameter of the inhibition zone provided insight into the antimicrobial properties of the distinct hydrogel compositions. Remarkably, the PVA/SA/GO/GSH hydrogel exhibited superior antibacterial activity against *S. aureus*, as reflected in Figures 5G, H. This finding underscores the potential of this hydrogel formulation as an effective antimicrobial agent in wound care.

### 3.6 Hydrogel-mediated wound healing assay *in vivo*

A full-thickness skin wound model in diabetic mice was utilized to ascertain the therapeutic potential of the hydrogels on chronic diabetic wounds. Diabetes was induced using Streptozotocin (STZ), and its successful establishment was confirmed through monitoring weight and fasting blood glucose fluctuations (Supplementary Figure S5). Following diabetes confirmation, full-thickness skin excisions simulated diabetic ulcer conditions in these mice. The mice were subsequently categorized into four groups: three treated with distinct hydrogel dressings and one untreated Control. The dimension of the wound was meticulously measured using digital calipers, noting both length and breadth (Supplementary Figure S6).

**FIGURE 6:**
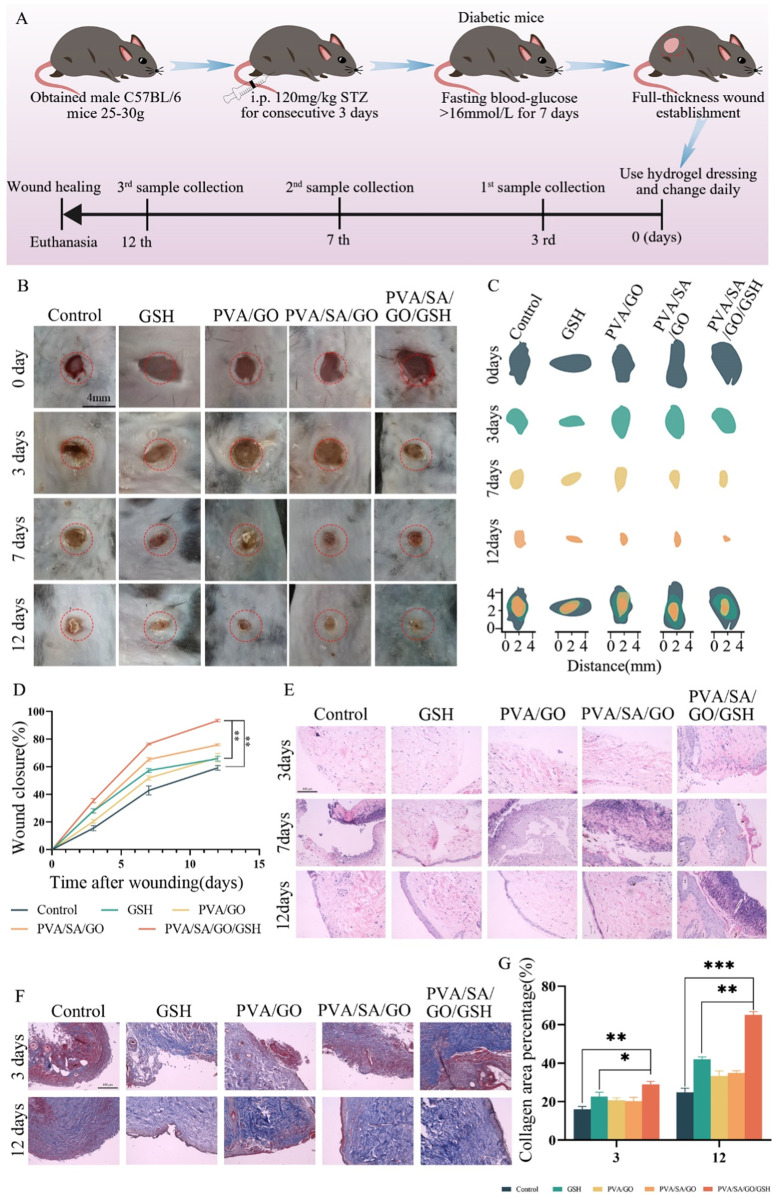
Comprehensive Evaluation of Hydrogel Efficacy in Diabetic Wound Healing. **(A)** Schematic illustration outlining the diabetic wound establishment in mice, including the initiation of the diabetes model, wound creation, and subsequent hydrogel application regimen. **(B)** Chronological photographic representation of wound healing under various treatment conditions, captured on days 0, 3, 7, and 12. **(C)** Schematic diagrams delineating wound closure dimensions across the evaluated time frame for each treatment group. **(D)** Graphical representation detailing the percentage wound closure over 12 days post-wounding, comparing different treatment methodologies. **(E)** Histological analysis through Hematoxylin and Eosin (H&E) staining from tissue samples across different treatment cohorts, observed on days 3, 7, and 12 post-wounding. **(F)** Masson’s Trichrome staining of wound sections on days 3 and 12, offering insights into collagen distribution and tissue architecture. Scale bar is 100 μm. **(G)** Quantitative evaluation, indicating the collagen area percentage Data are presented as means ± standard deviation. Statistically significant differences are denoted by asterisks, with *p ≤ 0.05, **p ≤ 0.01.

The PVA/SA/GO/GSH hydrogel and GSH groups demonstrated notably enhanced wound closure rates when compared to other groups (Figures 6B, C). Specifically, by day seven, the PVA/SA/GO/GSH group exhibited an 80% wound closure rate, nearing full wound recovery by day twelve. In contrast, alternative treatments yielded more prolonged healing intervals, with the Control group failing to achieve full recovery within the twelve-day timeframe. Quantitative data (Figure 6D) showcased an accelerated healing rate in all treatment groups relative to the Control, with PVA/SA/GO/GSH hydrogel significantly surpassing the GSH group.

The PVA/SA/GO/GSH hydrogel’s efficacy is likely due to its ability to maintain a moist wound milieu. This hydrogel further ensures minimal tissue adhesion and, thus, can be removed effortlessly without causing additional injury or discomfort.

Histological analyses via hematoxylin and eosin (HE) staining (Figure 6E) were performed to investigate tissue-level changes. Initial observations on day three revealed inflammation across all groups, yet the PVA/SA/GO/GSH hydrogel group indicated initial signs of tissue repair. By day seven, distinctions between groups became pronounced, with the PVA/SA/GO/GSH hydrogel group nearing complete recovery. By day twelve, the same group exhibited a full restoration, indistinguishable from surrounding healthy tissues. The overall histological findings highlight the PVA/SA/GO/GSH hydrogel’s ability to promote comprehensive wound repair.

To evaluate collagen distribution and organization, wound tissues underwent Masson’s Trichrome staining on days three and twelve (Figures 6F, G). The findings revealed superior collagen deposition and organization in the PVA/SA/GO/GSH group, highlighting its potential in enhancing structural components of wound healing.

In conclusion, the PVA/SA/GO/GSH hydrogel exhibits remarkable potential in addressing diabetic-induced wound challenges, paving the way for potential clinical applications.

### 3.7 Hydrogel-mediated antioxidant and antiglucose effects *in vivo*

**FIGURE 7:**
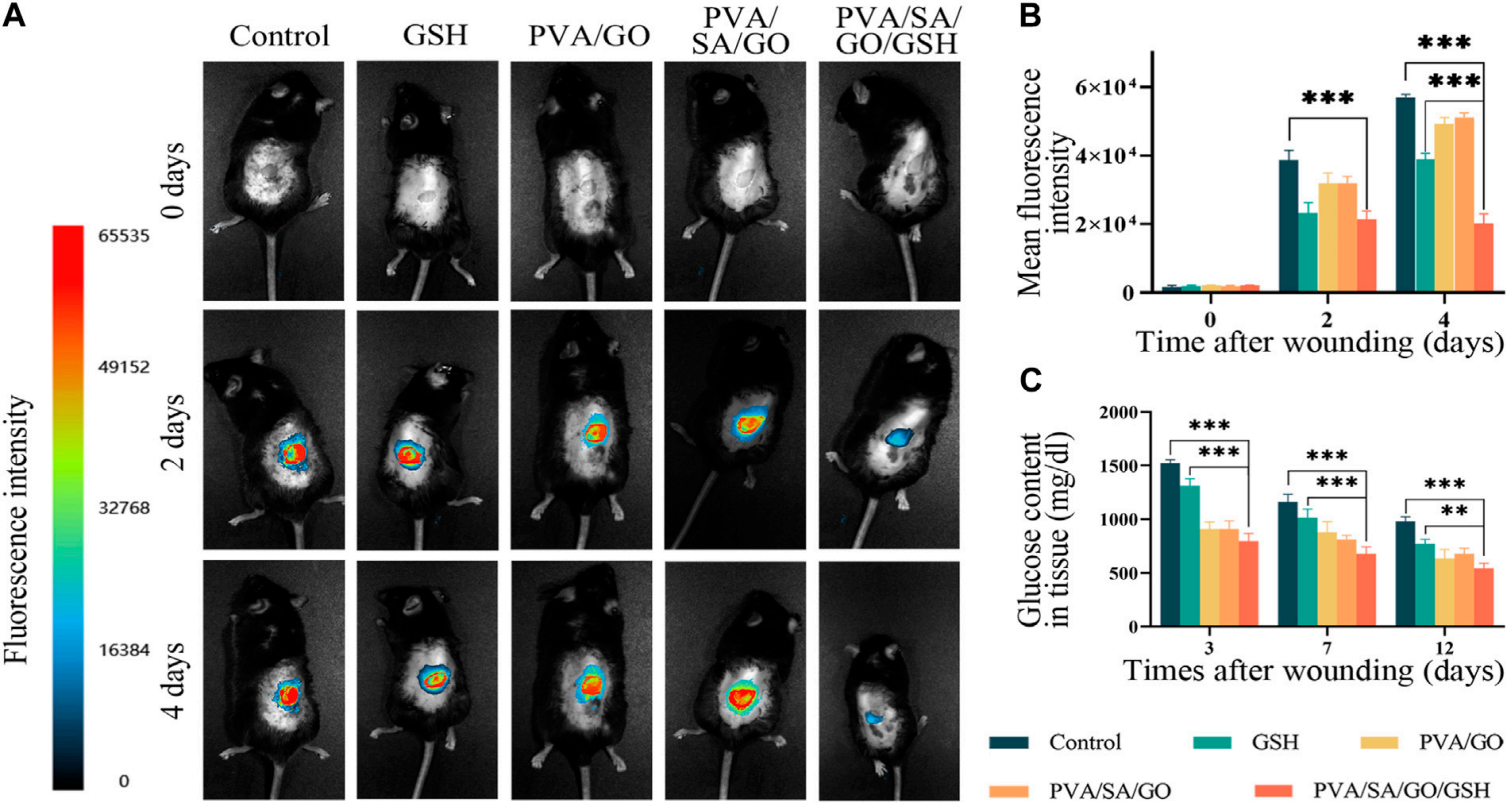
Impact of hydrogel treatment on wound healing and reactive oxygen species (ROS) scavenging. **(A)** Representative fluorescence images of wounds from day 0 to day 4; **(B)** Statistical analysis of average fluorescence intensity levels. **(C)** Quantitative analysis of glucose content in the wound tissues. Data are presented as means ± standard deviation. Statistically significant differences are denoted by asterisks, with **p ≤ 0.01, ***p ≤ 0.001.

To ascertain the reactive oxygen species (ROS) concentration within the wounded area, a 100 μM solution of 2′,7′-dichlorofluorescin diacetate (DCFH-DA) was utilized as a fluorescent probe (Zhang et al., 2022). Following its application to the wound site, fluorescence images were captured and subsequently, the mean fluorescence intensity was quantified (Figures 7A, B). At day 0, no fluorescence was detected in any group, which can be attributed to the wound being in the hemostasis phase and the inflammatory cells not yet having infiltrated the wound site. By the second day, all groups exhibited a significant upsurge in ROS levels. A steady increase in fluorescence intensity was observed in the Control group at day 4, suggestive of prolonged inflammation as a consequence of diabetes. The PVA/SA/GO/GSH hydrogel group and the GSH group, both containing GSH, demonstrated notable ROS scavenging capacity. The PVA/SA/GO/GSH hydrogel-treated group exhibited slightly lower ROS levels compared to the GSH group, potentially resulting from the sustained release of GSH from the hydrogel and the consumption of ROS by the cyclo-boron ester bond, as elaborated in Figure 5E.

To delve deeper into the implications of oxidative stress, Malondialdehyde (MDA), Superoxide Dismutase (SOD), and ROS levels in the wound tissue were quantified. The levels of MDA, indicative of the degree of oxidative damage to tissues, were found to be relatively elevated in the Control group due to the diabetic condition (Supplementary Figure S7A). However, all treated groups exhibited reduced MDA levels. SOD, a key endogenous antioxidant, showed increasing levels in the PVA/SA/GO/GSH hydrogel-treated group during the course of treatment, significantly exceeding the levels in both the Control and GSH groups (Supplementary Figure S7B). Furthermore, the ROS levels in the tissue derived from the wound site displayed a decrease with the progression of treatment, with the PVA/SA/GO/GSH hydrogel treatment group recording the most rapid decline in ROS concentrations (Supplementary Figure S7C). These observations underscore the ability of the PVA/SA/GO/GSH hydrogel to effectively mitigate oxidative stress, thereby promoting wound healing.

Concurrently, glucose levels in the injured tissues were measured via the O-toluidine method (Figure 7C). The results revealed high glucose levels in all groups on the third post-injury day, which showed a decline following PVA/SA/GO/GSH hydrogel treatment, implying substantial glucose consumption associated with the cleavage of the cyclic boron ester bond.

### 3.8 Anti-inflammatory and regenerated effects of hydrogel on diabetic mice wounds

Inflammation plays a crucial role in the wound healing process with interleukin-6 (IL-6) (Tu et al., 2022) and tumor necrosis factor-alpha (TNF-α) (Gharaboghaz et al., 2020) serving as key cytokines in the inflammatory response. We assessed the influence of hydrogel treatment on the expression of IL-6 and TNF-α via Western blot and enzyme-linked immunosorbent assay (ELISA) methods.

**FIGURE 8:**
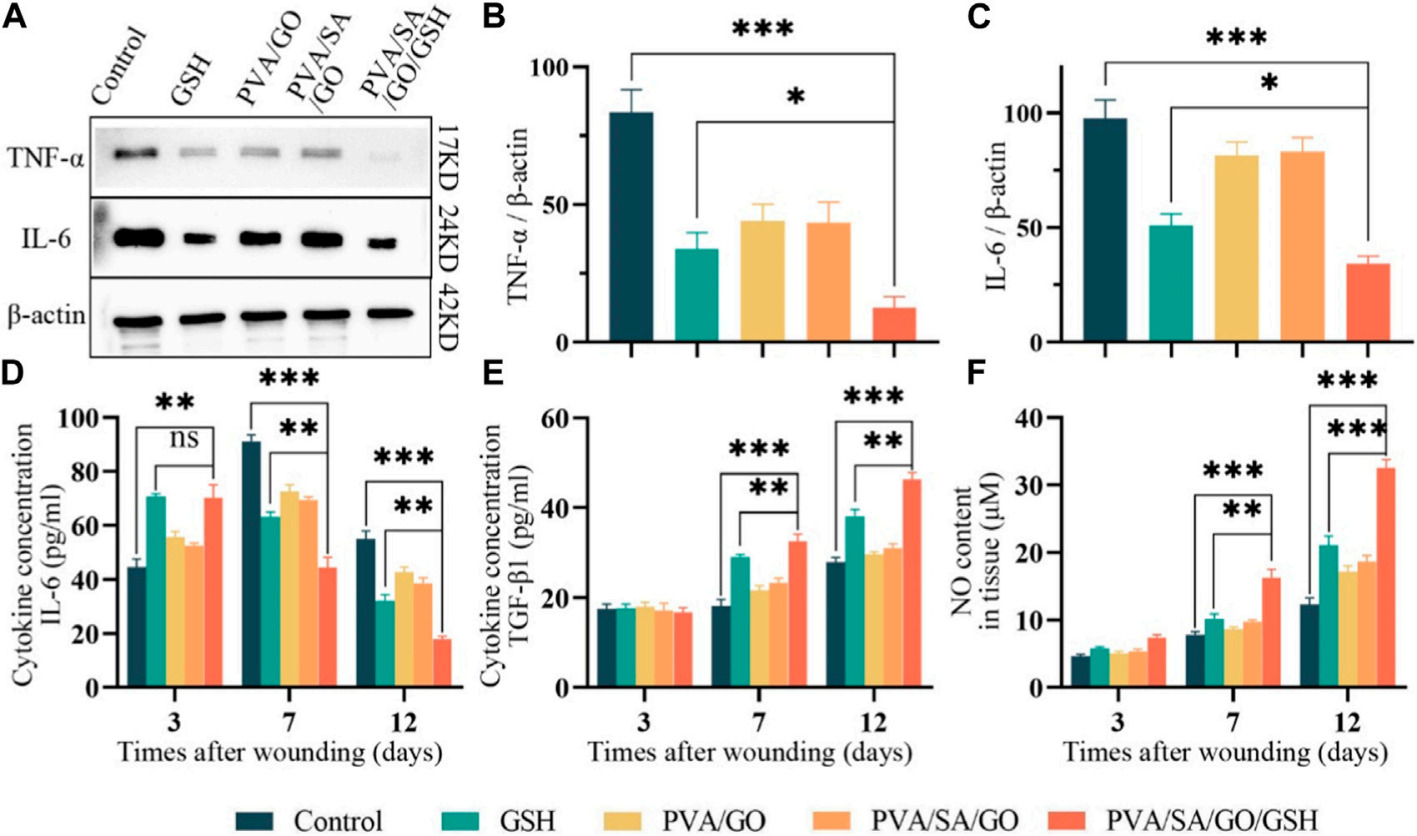
Evaluation of anti-inflammatory properties *in vitro* and *in vivo*. **(A)** Western blotting analysis used for the detection of IL-6 and TNF-α levels, with quantitative analyses of **(B)** IL-6 and **(C)** TNF-α levels. β-actin was employed as the internal control. Levels of **(D)** IL-6 and **(E)** TGF-β_1_, respectively, in the traumatized tissues as determined by enzyme-linked immunosorbent assay (ELISA). **(F)** Quantitative analysis of NO in wound tissues. Data are presented as means ± standard deviation. Statistically significant differences are denoted by asterisks, with *p ≤ 0.05, **p ≤ 0.01, ***p ≤ 0.001, whereas ‘ns’ signifies no significant difference.

Both the PVA/SA/GO/GSH hydrogel and GSH groups exhibited a marked reduction in TNF-α and IL-6 levels, as evidenced by the Western blot results (Figures 8A–C). Furthermore, ELISA analysis unveiled that IL-6 levels significantly escalated on the third day post-surgery in both the GSH group and the PVA/SA/GO/GSH group relative to the Control group (Figure 8D).

Nevertheless, by day 7, a striking increase in IL-6 levels was observed in the Control group, while the treatment groups displayed substantially reduced levels. As wound healing progressed, IL-6 levels witnessed a decline in all groups, with stabilization achieved by the 12th postoperative day.

It is noteworthy that the PVA/SA/GO/GSH hydrogel and GSH groups manifested a swifter ascent in IL-6 levels during the initial 3 days, succeeded by a significant plunge during the treatment course. This suggests that the treatment considerably mitigated the inflammatory response and abbreviated the inflammatory phase (Figure 8D). These observations align with the Western blot findings, thereby corroborating the anti-inflammatory efficacy of the hydrogel treatment.

TGF-β_1_, a pivotal factor regulating cellular functions including growth, differentiation, proliferation, migration, and apoptosis during wound healing, was analyzed through ELISA (Budi et al., 2019). Our findings indicated a uniform low concentration of TGF-β_1_ across all groups on the third post-operative day, suggesting a persistent inflammatory phase. However, by day 7, TGF-β_1_ concentrations increased in all groups, with the hydrogel PVA/SA/GO/GSH and GSH groups registering a statistically significant surge compared to the control group. By day 12, TGF-β_1_ concentrations saw a significant escalation in the treatment groups, especially in the PVA/SA/GO/GSH hydrogel group (Figure 8E).

We investigated nitric oxide (NO) levels - a critical mediator for endothelial cell function that is often diminished in trauma - using the Griess Reagent method (Kwon et al., 2019; Du et al., 2022). Results revealed that the PVA/SA/GO/GSH hydrogel effectively enhanced NO content in trauma tissues, thereby improving endothelial cell functions. Interestingly, the NO enhancement was more pronounced with the PVA/SA/GO/GSH hydrogel compared to the free GSH group (Figure 8F), attesting to the superiority of the dosage form in wound healing promotion.

### 3.9 Hydrogel’s effect on VEGF expression and neovascularization

**FIGURE 9:**
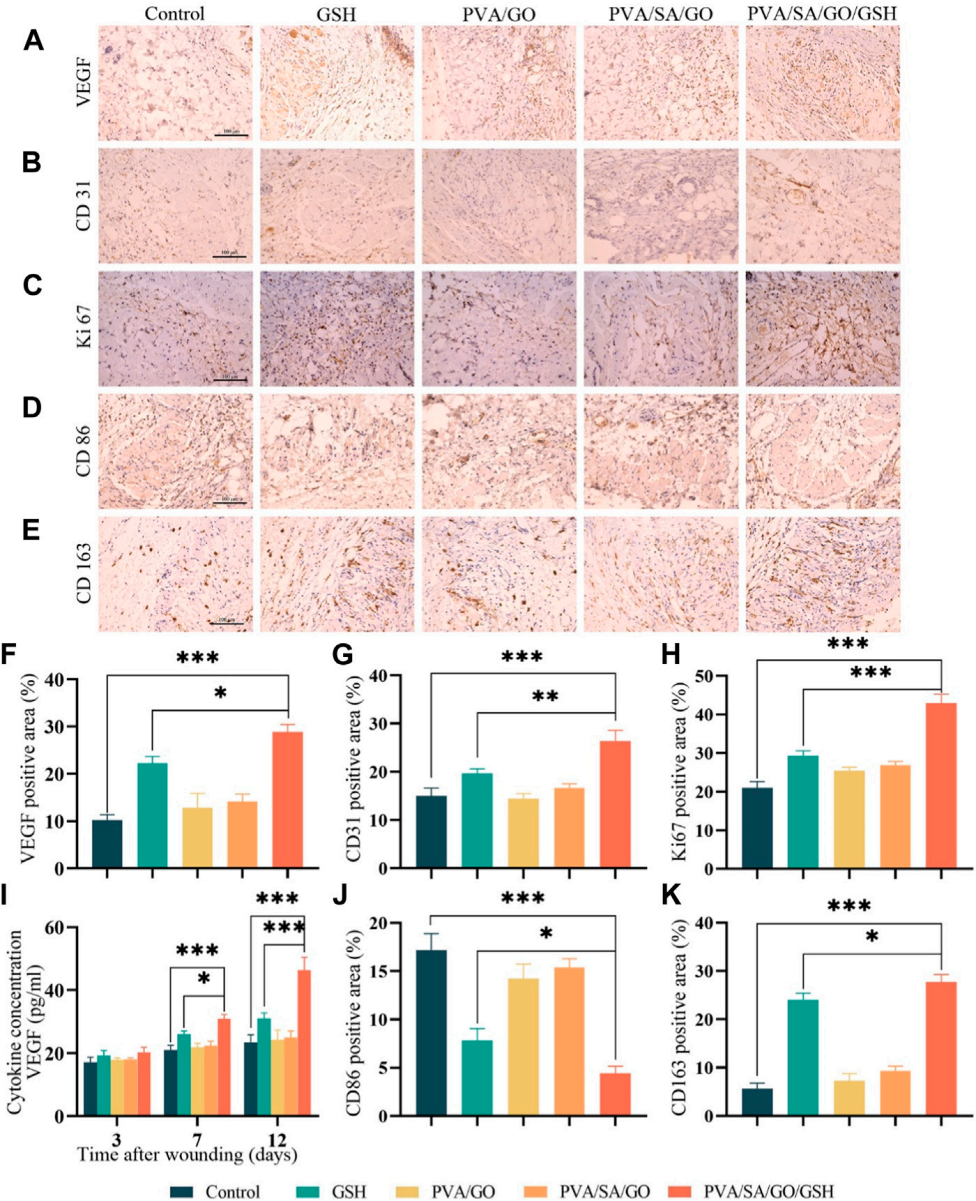
The effects of hydrogel on the microenvironment of the wound and the promotion of the healing of chronic diabetic wounds. Immunohistochemical staining was performed to detect the expression of **(A)** VEGF, **(B)** CD31, **(C)** Ki67, **(D)** CD86, and **(E)** CD163 (positive staining is brownish, nucleus staining is purple). Scale bar is 100 μm. Quantitative analysis of **(F)** VEGF, **(G)** CD31, **(H)** Ki67, **(J)** CD86, and **(K)** CD163 immunohistochemical staining was also performed. **(I)** ELISA was used to detect the expression of VEGF in the traumatic tissues. Data are presented as means ± standard deviation. Statistically significant differences are denoted by asterisks, with *p ≤ 0.05, **p ≤ 0.01, ***p ≤ 0.001.

VEGF plays a vital role in the process of wound healing by regulating cell proliferation, migration, and angiogenesis (Gharaboghaz et al., 2020), and its deficiency is one of the main causes of delayed wound healing in diabetic patients, which leads to reduced neovascularization. To investigate the effect of the hydrogel on VEGF expression, we performed immunohistochemistry staining and ELISA experiments. The staining images showed that the expression levels of VEGF in the hydrogel PVA/SA/GO/GSH treatment and GSH groups were significantly higher than the Control group, with the hydrogel PVA/SA/GO/GSH treatment group showing a much higher expression of VEGF than the GSH group (Figures 9A, F). The ELISA results were consistent with the immunohistochemistry staining, with the hydrogel PVA/SA/GO/GSH treatment group showing a significant increase in VEGF expression compared to the Control and GSH groups (Figure 9I). These findings suggest that the hydrogel PVA/SA/GO/GSH can effectively upregulate the expression of VEGF and promote neovascularization during the wound healing process.

CD31 can indicate the level of angiogenesis, which is the process of new blood vessel formation (Chen et al., 2015). In Figures 9B, G, the hydrogel PVA/SA/GO/GSH group and the GSH group showed positive expression of CD31 compared to the Control group, indicating an increase in angiogenesis. The positive expression in the hydrogel PVA/SA/GO/GSH group was higher than that in the GSH group.

To detect cell proliferation in the local wound tissue, Ki67 immunohistochemical staining was performed on skin wound tissue sections on postoperative day 7. Abundant Ki67 expression was detected in each treatment group, while in the Control group only a few Ki67-positive cells were detected, with statistically significant differences (Figures 9C, H). This indicates that the hydrogel PVA/SA/GO/GSH treatment promotes cell proliferation and may contribute to the accelerated healing of diabetic chronic wounds.

Macrophage phenotype is an essential indicator to evaluate wound healing progress. During this process, the transformation of macrophages from the hyperinflammatory M1 to the anti-inflammatory M2 phenotype plays a crucial role (Boniakowski et al., 2017). M1 macrophages release excessive inflammatory chemokines and pro-inflammatory cytokines, leading to persistent inflammation, while M2 macrophages promote tissue remodeling, angiogenesis, and wound healing (Eming et al., 2017). To observe M1 and M2 macrophages in the injury area, we performed immunohistochemical staining of CD86 and CD163 on skin wound tissue sections. The results showed that CD86-positive cells gradually decreased in the treatment group, and the hydrogel PVA/SA/GO/GSH treatment group had the least CD86-positive cells, which was significantly different from the GSH group (Figures 9D, J). On the other hand, the positive staining of CD163 gradually increased in the treatment group, with the hydrogel PVA/SA/GO/GSH treatment group showing the highest positive staining, which was also significantly different from the GSH group (Figures 9E, K). These findings indicate that the hydrogel PVA/SA/GO/GSH treatment promotes the transformation of macrophages from the M1 phenotype to the M2 phenotype, which helps in promoting wound healing.

Collectively, these findings underscore the PVA/SA/GO/GSH hydrogel as a potent therapeutic modality, optimizing the intricate balance of angiogenesis, cell proliferation, and immunomodulation, thereby expediting diabetic wound healing.

### 3.10 Evaluating in vivo safety of hydrogels

To ascertain the potential irritant response elicited by the biomaterials, skin irritation experiments were undertaken. Data from these experiments, as outlined in Supplementary Table S2, revealed negligible differences across the Control and GSH groups, PVA/GO group, PVA/SA/GO group, and the PVA/SA/GO/GSH group. This implies the biomaterials did not induce any discernible irritation in the organisms. Further, a comprehensive examination of the dorsal skin of the mice from each group indicated an absence of erythema or edema. These observations suggest that the biomaterials used are devoid of any significant skin irritation properties, reinforcing their potential applicability in wound healing procedures.

**FIGURE 10:**
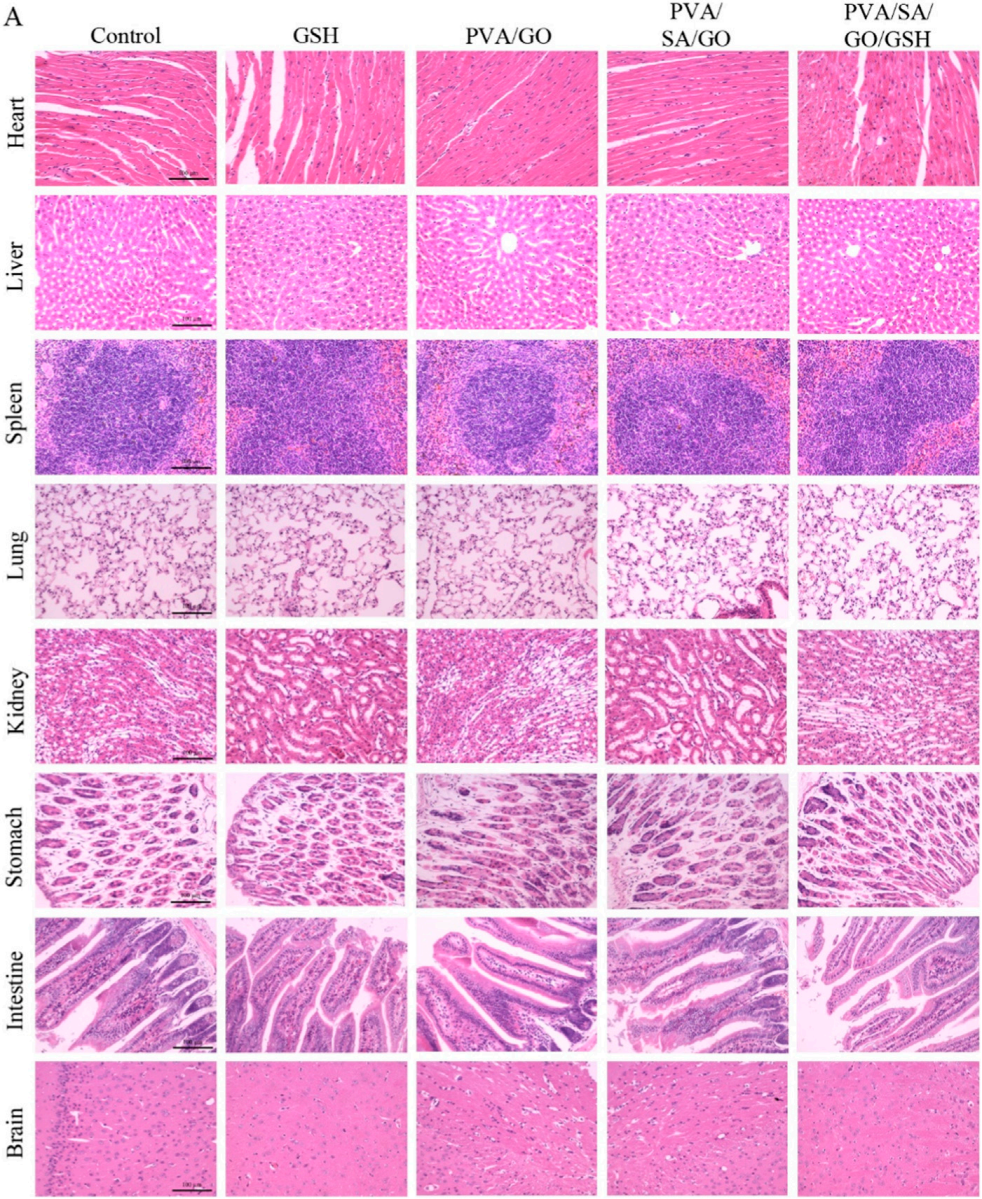
The histological examination of major organs such as the heart, liver, spleen, lung, kidney, stomach, intestine, and brain in diabetic mice treated with each group of hydrogels. H&E staining was performed, and the images were captured under a microscope. The scale bar is 100 μm.

An integral aspect of assessing the *in vivo* safety of the hydrogels encompassed a thorough histological examination of primary organs, including the heart, liver, spleen, lungs, kidneys, stomach, intestines, and brain, as depicted in Figure 10. The goal was to scrutinize any potential adverse impacts of the hydrogel treatment on the architecture of these organs. The findings demonstrated normal organ structures, implying that the hydrogel treatment did not trigger significant detrimental effects on these organs. These observations underscore the safety profile of the hydrogel treatment in a live organism, fortifying its potential application in medical procedures.

## 4 Discussion

In the early stages of diabetic wound healing, reactive oxygen species (ROS) levels are elevated without an increase in antioxidant enzyme activity, which would lead to a significant increase in oxidative stress in the injury microenvironment. Of particular concern is that persistent hyperglycemia in diabetic wounds directly leads to excessive accumulation of ROS (Xu et al., 2020), which disrupts the physiological environment and further slows the healing process (Tyeb et al., 2020). Therefore, biomaterials with the ability to modulate the inflammatory response, inhibit ROS overaccumulation, deplete glucose, and promote angiogenesis contribute to the healing of diabetic ulcers.

It has been shown that graphene oxide (GO) has a unique layered structure and abundant -OH groups, giving GO the potential to be used as a slow-release drug carrier (Li et al., 2019). Meanwhile, glutathione (GSH) acts as an antioxidant to scavenge excess ROS and can modulate oxidative stress on traumatic surfaces (Kwon et al., 2019). Therefore, in the present study, we developed a polyvinyl alcohol/sodium alginate/graphene oxide/glutathione (PVA/SA/GO/GSH) hydrogel with antioxidant and anti-inflammatory properties.

Supplementary Figure S1 presents a quantitative assessment of cell viability in response to graphene oxide (GO) and glutathione (GSH) exposure. Supplementary Figure S1A demonstrates that L929 cell viability is not significantly affected by lower concentrations of GO but shows a decrease at higher concentrations, indicating potential cytotoxic effects at these levels. Conversely, Supplementary Figure S1B indicates that GSH positively influences L929 cell viability, with no significant decrease even at higher concentrations, suggesting good biocompatibility. These findings underscore the selective cytotoxicity of GO and the biocompatible nature of GSH, which are critical considerations in the design of our PVA/SA/GO/GSH hydrogel for diabetic ulcer treatment.

While the antioxidant glutathione (GSH) is known for its ability to diminish reactive oxygen species (ROS) at injury sites, traditional drug delivery methods have fallen short in maintaining the prolonged efficacy of GSH. To address this issue, we incorporated the principles of bioorthogonal click chemistry, facilitating the cross-linking of graphene oxide (GO) and boronic acid derivatives via cyclic boron ester bonds. We validated this cross-linking process using hydrogen nuclear magnetic resonance spectroscopy (1H-NMR) and fluorescein isothiocyanate (FITC), as delineated in Figure 1.

An examination using scanning electron microscopy (SEM) disclosed that the hydrogels exhibited a unique three-dimensional porous structure. The physical attributes of the hydrogels, including their water retention, swelling, and electrical conductivity, were assessed, yielding favorable results (Supplementary Figure S4), as demonstrated in Figure 2.

Upon induction with either glucose or H_2_O_2_, the PVA/SA/GO/GSH hydrogel showcased a responsive release pattern (Figures 3F, G), signifying that the cyclic boron ester bond is sensitive to glucose and H_2_O_2_. In the presence of either of these substances, the bond disintegrates, triggering hydrogel dissociation (Figure 3). This responsive mechanism ensures the sustained presence of therapeutic levels of GSH at the injury site.

The synthesized PVA/SA/GO/GSH hydrogels exhibited impressive biocompatibility as evidenced by hemolysis analysis and cytocompatibility assays. These tests demonstrated the hydrogels’ excellent compatibility with biological tissues. *In vitro* studies further demonstrated that PVA/SA/GO/GSH hydrogels effectively inhibited apoptosis (Figure 4D) and significantly enhanced the migration of L929 cells (Figures 5A, C). Finally, the PVA/SA/GO/GSH hydrogels demonstrated remarkable ROS scavenging ability *in vitro* (Figure 5E). GSH’s inherent antioxidant properties, coupled with the ROS-reactive and ROS-breaking capabilities of the cyclic borate ester bond, collectively enhanced the overall ROS scavenging effect, thereby substantiating the potential therapeutic applicability of these hydrogels.

In terms of antimicrobial activity, the PVA/SA/GO/GSH hydrogel was observed to significantly suppress *Staphylococcus aureus* (Figure 5G), a primary pathogen implicated in diabetic wound infections. This finding implies the potential of PVA/SA/GO/GSH hydrogel in curtailing the risk of diabetic wound infections and subsequent ulcer development.

These *in vitro* findings suggest that smart PVA/SA/GO/GSH hydrogels are expected to be ideal wound dressing candidates to address the problem of excessive ROS in chronic diabetic wounds due to their excellent biocompatibility and antioxidant properties.

After application to diabetic cortical trauma, PVA/SA/GO/GSH hydrogel was effective in removing ROS from the ulcer surface (Figure 7). The *in vivo* experiments demonstrating wound healing efficacy indirectly suggest the residue-free nature of the hydrogel. The wound photos post-treatment display clean and clear healing progress, implying that the hydrogel does not leave residues that impede wound healing. For more precise quantification, we measured MDA, SOD, and ROS levels after treatment of traumatized tissues at different time points (Supplementary Figure S7) MDA is an index used to indicate the extent of oxidative damage in wounds (Gharaboghaz et al., 2020), while SOD is an antioxidant enzyme widely present in organisms and plays a crucial role in their oxidative and antioxidant balance (Lv et al., 2020). In diabetic wounds, persistent hyperglycemia and infection lead to excessive accumulation of ROS, while activation of the immune system also produces large amounts of ROS, resulting in a significant increase in oxidative stress in the traumatized tissue (Xu et al., 2020). Due to the microenvironmental changes caused by diabetes, we observed a gradual decrease in MDA levels and a gradual increase in SOD levels with wound healing; however, compared to the control group, MDA levels were lower and SOD levels tended to increase more rapidly in all treated groups. In addition, ROS levels decreased with increasing treatment time. These results suggest that the reduction in oxidative stress may be due to the antioxidant effect of GSH. Therefore, we suggest that the integration of GSH into wound dressings can effectively scavenge ROS, improve metabolism, and promote ulcer healing.

Control of inflammation is essential to promote chronic wound healing (Boniakowski et al., 2019). TNF-α mediates and amplifies the inflammatory response in traumatized tissues, drives cell migration, and is tightly associated with the healing of damaged tissues (Ritsu et al., 2017). IL-6 is an important pro-inflammatory cytokine that is released during infection or tissue injury (Tu et al., 2022). TNF-α and IL-6 are usually associated with inflammatory responses, and in each treatment group, their expression levels were lower than in the control group.

Macrophage phenotype is a key indicator to assess inflammation and proliferation, and different phenotypes of macrophages are closely associated with the onset, persistence, and end of inflammation (Oishi and Manabe, 2018). In an attempt to understand the dynamics of macrophage phenotypes within wounded tissues, we performed immunohistochemical staining targeting CD86 and CD163, representing M1 and M2 macrophages, respectively. Our analysis revealed an abundance of CD86-positive cells within the control wounds, suggesting a substantial infiltration of pro-inflammatory M1 macrophages, and consequently a heightened inflammatory response. This predominance of M1 macrophages can be attributed to the high-glucose environment, which impedes the phenotypic transition of macrophages, leading to an abnormal accumulation of M1 type.

Interestingly, upon treatment with GSH and PVA/SA/GO/GSH hydrogel, a significant decline in the M1 macrophage population was observed (Figure 9D). This decrease can be largely attributed to the potent anti-inflammatory properties of GSH. Moreover, supporting evidence from previous Western blot studies, demonstrating a correlation between reduced levels of reactive oxygen species (ROS) and the pro-inflammatory cytokine TNF-α, and a decrease in M1 macrophages, reinforces this finding. Collectively, these results underscore the ability of GSH and PVA/SA/GO/GSH hydrogel to modulate macrophage dynamics, thereby potentially alleviating the inflammatory response in wounds (Canton et al., 2021).

Notably, the transition from M1 macrophages to the M2 phenotype is the defining event in the transition from the inflammatory to the proliferative phase. M2 phenotype macrophages promote tissue repair, enhance angiogenesis, and stimulate collagen deposition within the wound by maintaining a balance of anti-inflammatory and pro-inflammatory (Boniakowski et al., 2017; Li et al., 2021). Our findings show a substantial increase in the M2 macrophage count in the group treated with PVA/SA/GO/GSH hydrogel compared to the other groups (Figure 9E). This suggests that PVA/SA/GO/GSH hydrogel can effectively stimulate the phenotypic transition of macrophages from the pro-inflammatory M1 phenotype to the reparative M2 phenotype. By doing so, it promotes tissue repair and expedites the proliferative and remodeling phases of healing in traumatized cells.

This result aligns with our observations that the group treated with PVA/SA/GO/GSH hydrogel showed a diminished inflammatory response, reduced ROS levels, shorter recovery times, and superior healing quality (Figure 6). Thus, the advantageous effects of PVA/SA/GO/GSH hydrogel can be partially attributed to its ability to modulate macrophage phenotypic transition, thereby accelerating the healing process.

Further evaluation of tissue reconstruction was carried out by performing Masson’s staining to determine the collagen content in the regenerated skin tissue. As expected, collagen levels rose during the healing process across all groups (Figure 6G). However, both the GSH and PVA/SA/GO/GSH hydrogel groups showed higher collagen content compared to the control group, implying that the application of these treatments facilitated collagen secretion, which in turn accelerated wound healing and tissue reconstruction.

NO can induce increased vascular permeability and increase local blood flow (Du et al., 2022). The experimental results showed that PVA/SA/GO/GSH hydrogel contributed to increasing the synthesis of NO (Figure 8F). Glutathione’s antioxidant properties may help reduce oxidative stress, which can otherwise inhibit nitric oxide synthase activity. By mitigating oxidative stress, glutathione may indirectly promote NO production and release, enhancing wound healing. TGF-β_1_ (Budi et al., 2019) and VEGF (Zhao et al., 2021) have important roles in dermal wound repair processes. The highest concentrations of TGF-β_1_ (Figure 8E) and VEGF (Figure 9I) were found in the wound and surrounding skin tissues in the PVA/SA/GO/GSH hydrogel group.

In the results of immunohistochemical analysis, enhanced CD31 expression was observed (Figure 9G), confirming the role of PVA/SA/GO/GSH hydrogel in promoting angiogenesis. The active degree of cell proliferation is closely related to the rate of wound healing, and Ki67, a nuclear protein closely related to cell proliferation (Sun et al., 2020), was expressed in the PVA/SA/GO/GSH hydrogel group at a higher level than in the control group (Figure 9H), suggesting that hydrogel treatment accelerates cell proliferation and thus promotes wound healing.

Comprehensive *in vivo* experimental results showed that the treatment of diabetic ulcer injury with PVA/SA/GO/GSH hydrogel could effectively promote angiogenesis and wound healing, inhibit bacterial growth, reduce oxidative stress, modulate inflammatory response, and promote orderly collagen deposition, which together promote the healing of diabetic ulcer injury.

Our novel PVA/SA/GO/GSH hydrogel exhibits unique properties when compared to existing graphene oxide and glutathione-based hydrogels in the literature. While previous hydrogels, such as graphene oxide/polymer composites (Hu et al., 2015; Alam et al., 2016; Navaei et al., 2017) and chitosan/graphene oxide hydrogels (Nath et al., 2018), emphasize aspects like electrical conductivity, mechanical strength, and multifunctionality, our hydrogel is distinctively tailored for diabetic ulcer treatment. Its design focuses on bio-responsiveness to glucose and reactive oxygen species levels, a critical aspect in diabetic wound healing. This specificity in application and response mechanism sets the PVA/SA/GO/GSH hydrogel apart, offering a targeted approach in managing diabetic ulcers, a domain less explored in existing hydrogel technologies.

## 5 Conclusion

Our research successfully developed a bioorthogonal click chemistry hydrogel, designed to release glutathione in response to oxidative stress, exhibiting significant potential for treating diabetic ulcers. The hydrogel demonstrated potent antioxidant, biocompatible, and antibacterial properties, accelerating wound healing in in vivo models. This study underlines the hydrogel’s potential for clinical application, while future research may further enhance its therapeutic efficacy by incorporating other bioactive molecules and optimizing its synthesis and release kinetics.

## Data Availability

The original contributions presented in the study are included in the article/Supplementary Material, further inquiries can be directed to the corresponding author.
