# Corrigendum: Double cross-linked graphene oxide hydrogel for promoting healing of diabetic ulcers

**DOI:** 10.3389/fchem.2024.1393387

**Published:** 2024-03-22

**Authors:** Wenxu Liu, Yunfang Yang, Meiying Li, Jingxin Mo

**Affiliations:** ^1^ Lab of Neurology, The Affiliated Hospital of Guilin Medical University, Guilin, China; ^2^ School of Pharmacy, Guilin Medical University, Guilin, China; ^3^ Health Management Centre, The Second Affiliated Hospital of Guilin Medical University, Guilin, China; ^4^ Clinical Research Center for Neurological Diseases of Guangxi Province, The Affiliated Hospital of Guilin Medical University, Guilin, China

**Keywords:** bioorthogonal click, graphene oxide, dual network hydrogel, diabetic ulcer damage, glutathione

In the published article, there was an error in Figure 7. The corrected figure and its caption appear below.

**FIGURE 7:**
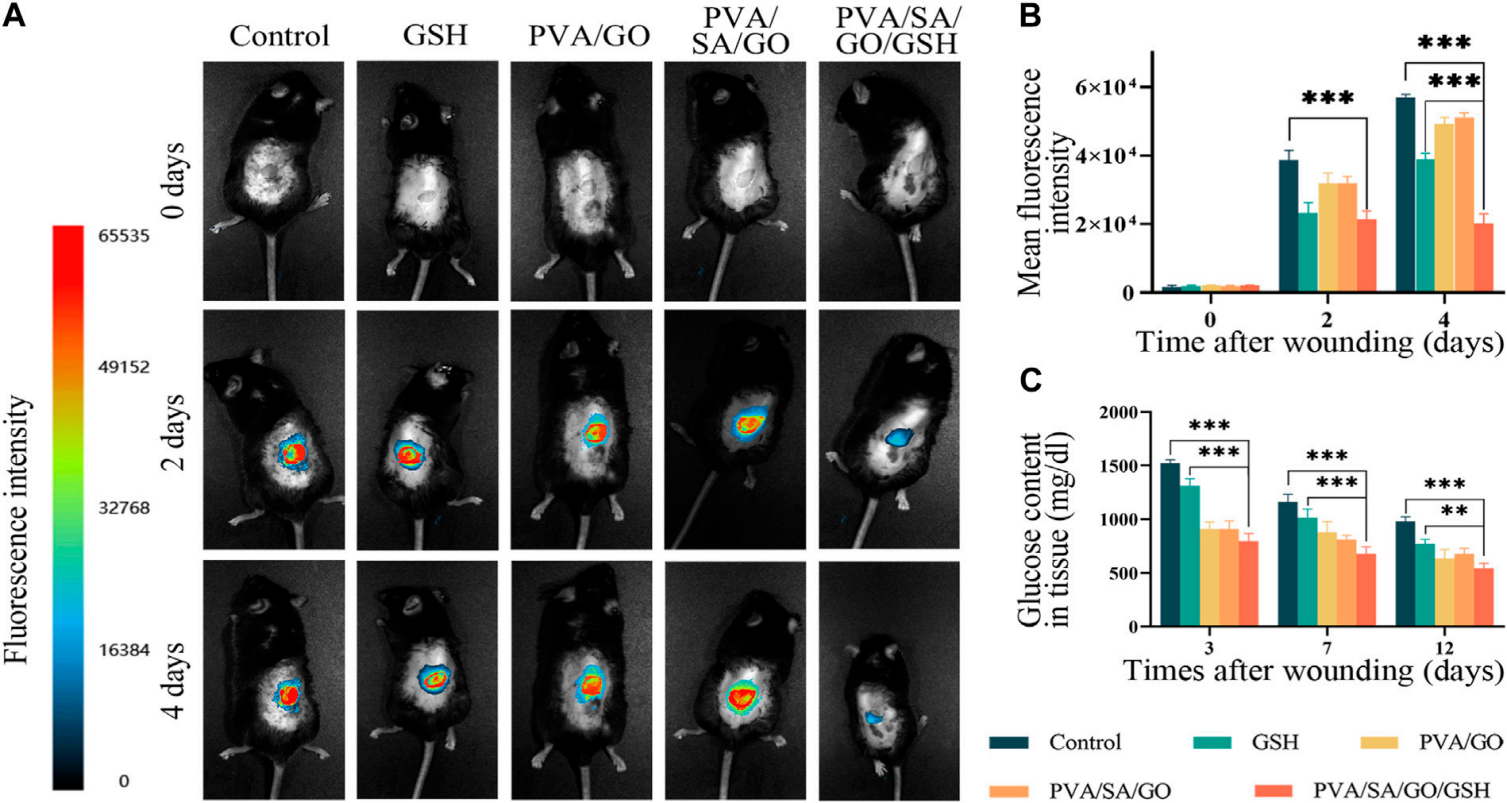
Impact of hydrogel treatment on wound healing and reactive oxygen species (ROS) scavenging. **(A)** Representative fluorescence images of wounds from day 0 to day 4; **(B)** Statistical analysis of average fluorescence intensity levels. **(C)** Quantitative analysis of glucose content in the wound tissues. Data are presented as means ± standard deviation. Statistically significant differences are denoted by asterisks, with **p ≤ 0.01, ***p ≤ 0.001.

In the published article, there was an error in Figure 9. The corrected figure and its caption appear below.

**FIGURE 9:**
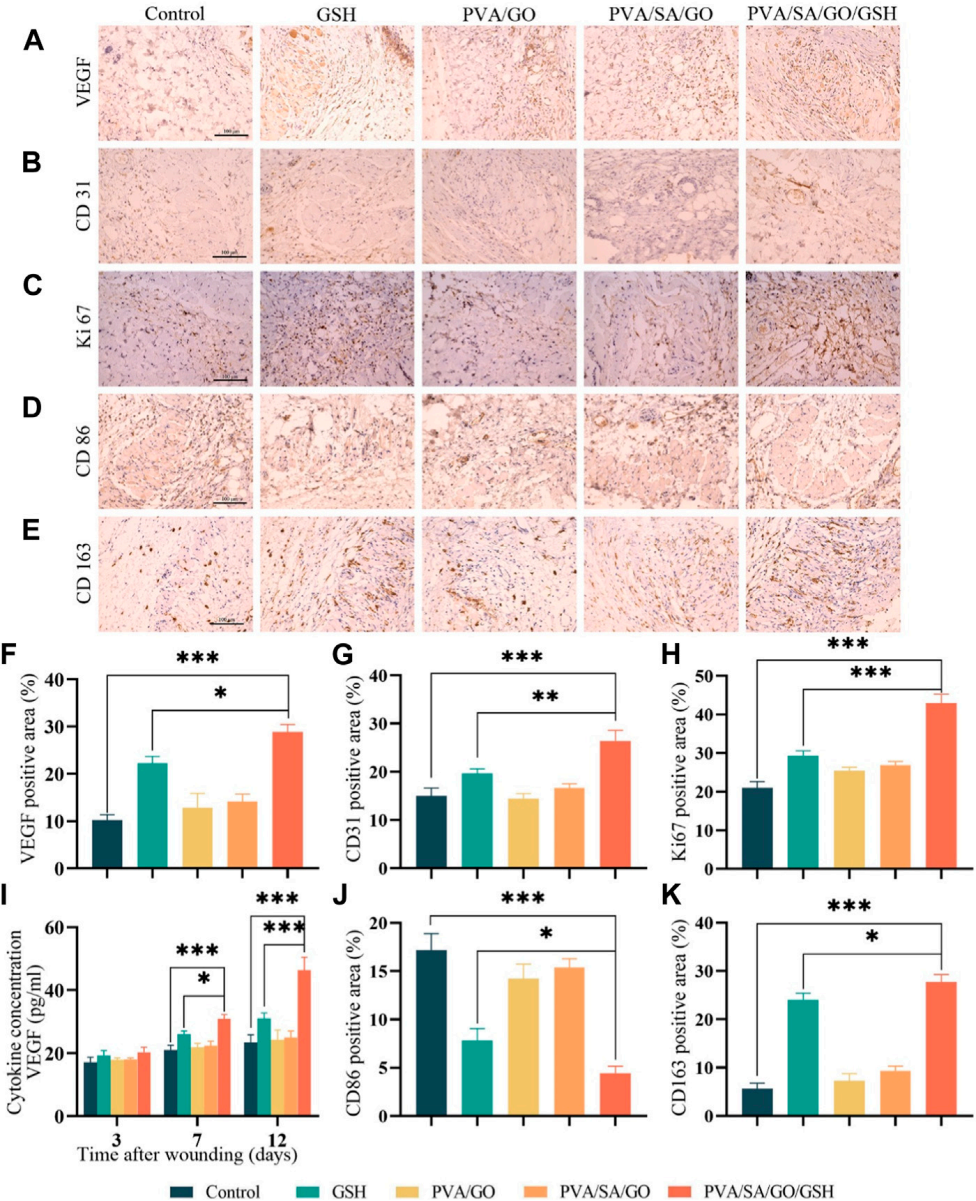
The effects of hydrogel on the microenvironment of the wound and the promotion of the healing of chronic diabetic wounds. Immunohistochemical staining was performed to detect the expression of **(A)** VEGF, **(B)** CD31, **(C)** Ki67, **(D)** CD86, and **(E)** CD163 (positive staining is brownish, nucleus staining is purple). Scale bar is 100 μm. Quantitative analysis of **(F)** VEGF, **(G)** CD31, **(H)** Ki67, **(J)** CD86, and **(K)** CD163 immunohistochemical staining was also performed. **(I)** ELISA was used to detect the expression of VEGF in the traumatic tissues. Data are presented as means ± standard deviation. Statistically significant differences are denoted by asterisks, with*p ≤ 0.05, **p ≤ 0.01, ***p ≤ 0.001.

The authors apologize for these errors and state that this does not change the scientific conclusions of the article in any way. The original article has been updated.

