# Supplementary material for: Double cross-linked graphene oxide hydrogel for promoting healing of diabetic ulcers
Source: Front Chem. 2024 Feb 22;12:1355646. doi: 10.3389/fchem.2024.1355646 (PMC10917884; doi:10.3389/fchem.2024.1355646)
Supplement: Supplementary file 1 [file DataSheet1.pdf]

Supporting Information

Novel glutathione-loaded Dual Network Hydrogel for Reducing Diabetic Ulcer Damage

Wenxu Liu[#1,2], Yunfang Yang[#3], Meiying Li[2], Jingxin Mo*[1,4]

[1] *Lab of Neurology, The Affiliated Hospital of Guilin Medical University, Guilin 541001, China*

[2] *School of Pharmacy, Guilin Medical University, Guilin 541001, China.*

[3] *Health Management Centre, The Second Affiliated Hospital of Guilin Medical University, Guilin 541001, China,*

[4] *Clinical Research Center for Neurological Diseases of Guangxi Province, The Affiliated Hospital of Guilin Medical University, Guilin 541001, China.*

[#]These authors have contributed equally to this work.

*Correspondent E-mail:

Jingxin Mo: Jingxin.mo@hotmail.com

Experimental details

Characterization

In order to determine the gel-forming time, the container was placed flat at regular intervals and the sol-gel transition was assessed by observing the deformation of the solution in the container. The time to reach the gel state was recorded as the gel time. To assess the structure of the lyophilized hydrogels, the cross sections were gold-sprayed and examined using scanning electron microscopy (SEM) at 5.00 kV. The water retention capacity of each group was determined by taking the same weight of hydrogel, drying it at 37℃, and weighing it every hour for 8 hours. The water retention ratio was then calculated using a specific formula.as follows:

$$\text{Water retention rate}(\%)=\frac{\text{hourly hydrogel mass}}{\text{initial hydrogel mass}}\times 100\%$$

The swelling property of the hydrogel was evaluated by gravimetric analysis. The weight of the samples was recorded periodically after they were immersed in physiological saline solution. The samples were removed and excess surface water was eliminated with filter paper. The initial weight (W_0) and the weight after swelling at various time points (Wt) were recorded to calculate the swelling ratio of the three groups of hydrogels. The swelling ratio was determined using the following formula:

$$\text{Swelling ratio}(\%)=\frac{W_t}{W_0}\times 100\%$$

For the measurement of electrical conductivity, stick electrodes were attached to both ends of the hydrogel sample, and the resistance was measured using an avometer. This test was performed three times for each sample, and the conductivity was calculated using the following formula: $\sigma = R/(L\times S)$, where σ is the conductivity in Siemens per meter (S/m), R is the resistance in ohms (Ω), L is the distance between the two electrodes in meters (m), and S is the cross-sectional area of the measured hydrogel in square meters (m^2).

Drug released

To assess the in vitro release characteristics of glucose in response, 8 g of PVA/SA/GO/GSH hydrogel containing glutathione (24 mg) was taken and divided into four tubes. Then, 40 ml of PBS with varying glucose concentrations of 0.0 mg/ml, 0.4 mg/ml, 1.0 mg/ml, and 3.0 mg/ml (referred to as GBS) was added to each tube, followed by shaking in a 37°C-water bath at specific intervals. At different time points, 3.5 ml of the supernatant was collected and the release profile of glutathione in different GBS was determined by UV-vis analysis at wavelengths of 230 nm, which corresponded to the maximum absorption of glutathione concentration.

For evaluating the cyclic release properties of the PVA/SA/GO/GSH hydrogel in response to changes in glucose concentration, 2 g of the hydrogel (containing 6mg of glutathione) was placed in a tube and 10 ml of 3.0 mg/ml GBS was added. The tube was incubated with shaking in a 37 ℃ water bath for 1h, and the supernatant was

collected and analyzed using UV-vis spectrophotometry. The buffer was then poured out, and the hydrogel was washed twice with PBS. The hydrogel was then placed in 0.4 mg/ml GBS, shaken in a water bath at 37℃ for 3 h, and the supernatant was collected. The process was repeated multiple times to evaluate the cyclic release properties.

For assessing the in vitro release characteristics of ROS in response, H_2O_2 concentrations of 0.00 mg/ml, 0.01 mg/ml, 0.05 mg/ml, and 0.10 mg/ml (referred to as HBS) were used. The hydrogel samples were incubated with the respective HBS solutions and shaken in a 37℃ water bath. The supernatant was collected at various time intervals and analyzed using UV-vis spectrophotometry to determine the release profile of ROS in different HBS solutions.

To evaluate the cyclic release properties of the hydrogel in response to changes in ROS concentration, a high concentration HBS solution (0.10 mg/ml) and a low concentration HBS solution (0.01 mg/ml) were chosen. The hydrogel was incubated with the high concentration HBS solution, and the supernatant was collected and analyzed using UV-vis spectrophotometry. The buffer was then poured out, and the hydrogel was washed twice with PBS. The hydrogel was then placed in the low concentration HBS solution, shaken in a water bath at 37℃ for 3 h, and the supernatant was collected. The cyclic release properties were evaluated by repeating this process multiple times.

In vitro biocompatibility

Hemolysis assay was conducted using freshly collected blood from BALB/c mice. The serum was removed from the blood and it was diluted with saline. The hydrogel sample

was then incubated in the erythrocyte suspension. Negative and positive controls were set up by adding the same amount of saline and TritonX-100 (1%), respectively. After incubation and centrifugation, the supernatant was collected and the absorbance was measured at 540 nm to determine the hemolysis rate. The hemolytic quantification was calculated using the following formula:

$$\text{Hemolysis ratio}(\%)=\frac{A_S\text{-}A_N}{A_P\text{-}A_N}\times 100\%$$

where AS represents the hydrogel group supernatant absorbance, AP represents the positive control absorbance, and AN represents the negative control absorbance.

For in vitro biocompatibility examination, the sterilized hydrogel was immersed in MEM culture solution and incubated at 37°C for 24 and 48 hours to obtain the extract. The extract was then centrifuged for later use. L929 cells were inoculated into a 96-well plate, and after cell adhesion, the medium was replaced with the extract and incubated for 24 hours. The cell viability was measured, and a control group without extract was set. The amount of GSH used in the GSH group was equivalent to the amount used in the hydrogel composition. The metabolic activity of cells treated with hydrogels was tested using the CCK-8 assay. The cell survival rate was calculated using the following formula:

$$\text{Cell survival rate}(\%)=\frac{\text{OD value of experiment group}}{\text{Control dialogue OD value}}\times 100\%$$

The cell scratch experiment was conducted by inoculating L929 cells in a 6-well plate at a density of 2×10^5 cells per well. After 24 hours of incubation to allow cell

attachment, a scratch was made in the cell monolayer using a sterile pipette tip. The cells were washed with PBS to remove the debris and incubated with serum-free hydrogel extract for 24h and 48h. The area of the scratch was measured using microscopy images taken at 0h, 24h, and 48h. The area reduction rate was calculated using the following formula:

$$\text{Area reduction rate}(\%)=\frac{\text{0h scratch area-48h scratch area}}{\text{0h scratch area}}\times 100\%$$

The Transwell experiment was conducted by first wetting the Transwell chambers with PBS, followed by adding 500 μl of MEM to the lower chamber and 200 μl of L929 cells (4×10^5 cells) to the upper chamber. The cells were incubated for 24h, and then the upper chamber was replaced with MEM containing hydrogel leachate. After 48h of further incubation, the cells were fixed with paraformaldehyde and stained with crystal violet. Random fields of view were selected and images were captured. The number of cells was counted using image j software.

In vitro antimicrobial capacity and ROS clear capacity

Assessment of in vitro antimicrobial capacity. Staphylococcus aureus (CMCCB26003) was cultured in a suitable medium. Solid medium was poured into a plate and an Oxford cup was added before it solidified. After spreading the bacterial solution mixed with solid medium evenly on both sides of the Oxford cups, the Oxford cups were removed after solidification. The hydrogel of each group was placed flat in the hole formed by

the Oxford cup and incubated. The formation of inhibition circles was observed, and the diameter was recorded and photographed.

Assessment of in vitro ROS clearing capacity. Hydrogel groups were incubated with L929 fibroblasts treated with ROS-up agent to stimulate excessive intracellular ROS production. Dichloro-dihydro-fluorescein diacetate (DCFH-DA) was used as a probe to detect ROS levels, and its fluorescence was activated by oxidation of DCFH-DA by intracellular ROS.

Establishment of the diabetic ulcer damage mouse model

Male BALB/c mice weighing 32 $\pm$ 2 g were randomly selected and fasted for 12 hours prior to modeling. On the day of modeling, their body weight was recorded, and blood glucose was measured after blood collection from the tail vein. The mice were then intraperitoneally injected with STZ at a dose of 120 μg/g body weight. Following the injection, the mice were provided with sufficient drinking water and food. Changes in the body weight and blood glucose levels of the mice were recorded for three consecutive days after the injection. Anesthetized mice were immobilized, and the dorsal hair was removed. The surgical site was sterilized using 75% ethanol. A circular, full-thickness skin wound with a diameter of 4 mm was created on the back using a sterile biopsy punch under aseptic conditions. In the case of active bleeding, sterile gauze packing or pressure was applied to stop the bleeding.

In vivo wound oxidative stress levels Assessment

The tissue was rapidly frozen in liquid nitrogen, and a lysis solution ten times the tissue mass was added. After sufficient lysis on ice, the tissue was centrifuged at 4°C for 10 minutes, and the resulting supernatant was collected.

MDA level was assessed by preparing the TBA storage solution and antioxidant into the MDA assay working solution. 0.1 ml of the sample was mixed with 0.2 ml of the MDA assay working solution, heated at 100℃ for 15 minutes, cooled to room temperature in a water bath, and then centrifuged at room temperature for 10 minutes. The resulting supernatant was added to a 96-well plate, and the absorbance was measured at 532 nm. The absorbance and MDA content were calculated using the standard curve and tissue weight.

SOD level was measured by mixing the supernatant of the tissue homogenate with the enzyme reaction solution, and incubating the mixture at 30℃ for 30 minutes. The absorbance was measured at 450 nm, and the inhibition rate of SOD activity and SOD enzyme activity units in the samples were calculated using a formula.

$$\text{Percent inhibition of SOD activity}=\frac{A_{\text{blank control 1}}-A_{\text{sample}}}{A_{\text{blank control 1}}-A_{\text{blank control 2}}}$$

$$\text{SOD enzyme activity units}=\frac{\text{Percent inhibition of SOD activity}}{\text{1-Percent inhibition of SOD activity}}\times 100\%$$

The blank control in SOD activity measurement refers to the absorbance of the enzyme reaction working solution and the reaction starter working solution after incubating the sample for the same time.

3. To determine the level of reactive oxygen species (ROS), the O13 ROS probe was used as a red-light fluorescent probe. The tissue supernatant and O13 probe were mixed

well and incubated for 20 minutes away from light. The intensity of tissue ROS was expressed as fluorescence intensity (RFU) per protein concentration (mg protein). For the determination of nitric oxide (NO) level, tissue supernatant was collected and the content of NO was measured by the Griess Reagent method.

Supplementary figures

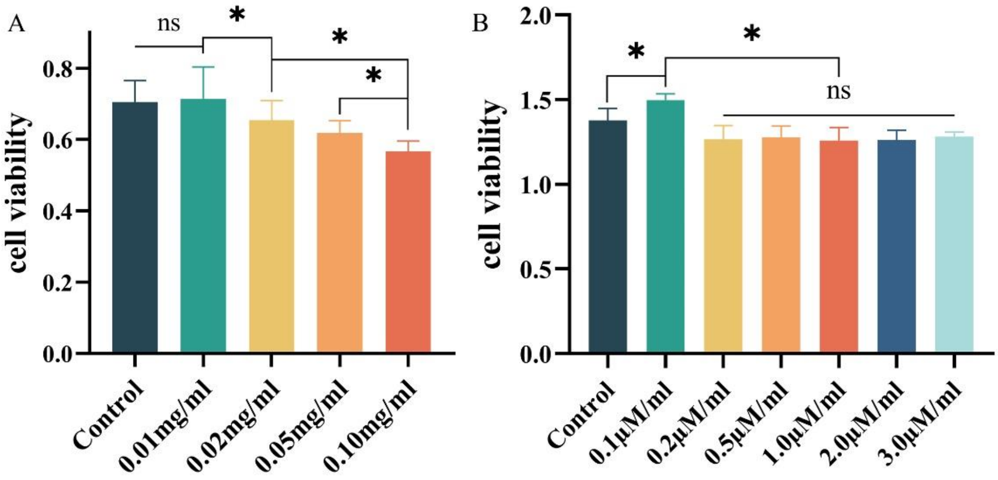

Figure S1: (A) The impact of graphene oxide (GO) on L929 cells viability, (B) The stimulatory effect of glutathione (GSH) on L929 cells viability. Data are presented as means ± standard deviation. Statistically significant differences are denoted by asterisks, with $*p \leq 0.05$, while 'ns' represents a non-significant difference.

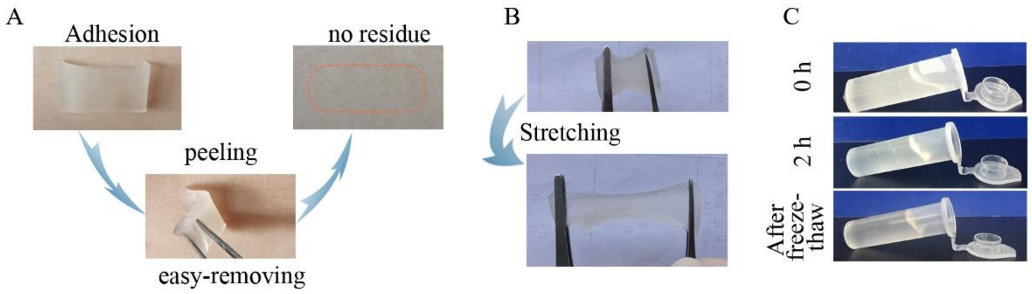

Figure S2. Comprehensive evaluations of hydrogel properties. (A) The hydrogel's efficient adherence to the skin and its subsequent residue-free removal, signifying its optimal adhesive capability. (B) The tensile resilience of the PVA/SA/GO/GSH hydrogel when subjected to external stretching forces. (C) The transformation of the hydrogel under freeze-thaw conditions, emphasizing its structural stability.

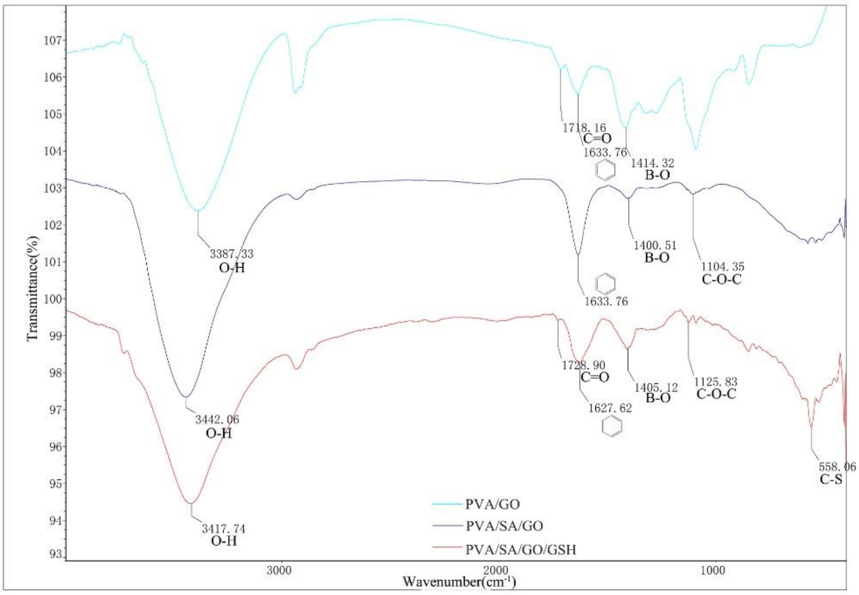

Figure S3. Fourier transform infrared (FTIR) spectra results of PVA/SA/GO/GSH hydrogels before and after crosslinking. The spectra showed characteristic peaks of each component, and the shift of the peaks indicated the interaction and crosslinking among the components.

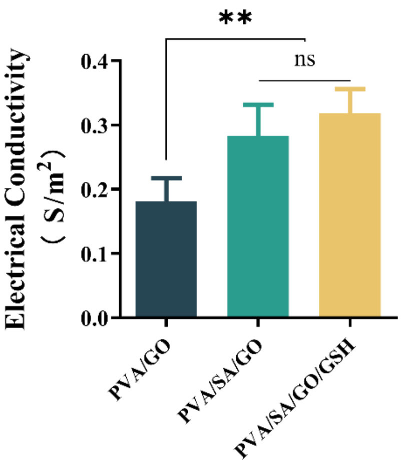

Figure S4. Electrical conductivity of the PVA/SA/GO/GSH hydrogels. The electrical conductivity of the hydrogels was measured using a two-point probe method. The results showed that the conductivity of the hydrogels increased with the increasing

content of GO, indicating that the addition of GO enhanced the conductive properties of the hydrogels. Data are presented as means ± standard deviation. Statistically significant differences are denoted by asterisks, with **$p \leq 0.01$, while 'ns' represents a non-significant difference.

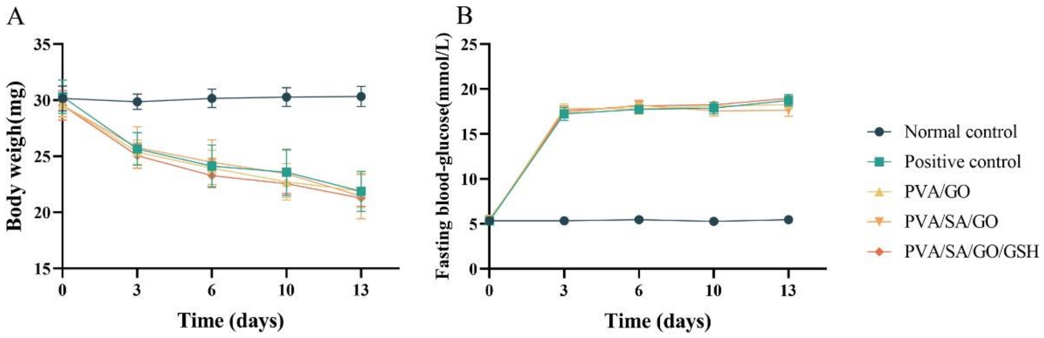

Figure S5. (A) The changes in body weight of mice during the modeling period were recorded, (B) The fasting blood glucose levels of mice during the modeling period were measured. The data are presented as means ± standard deviation (SD) of three independent experiments.

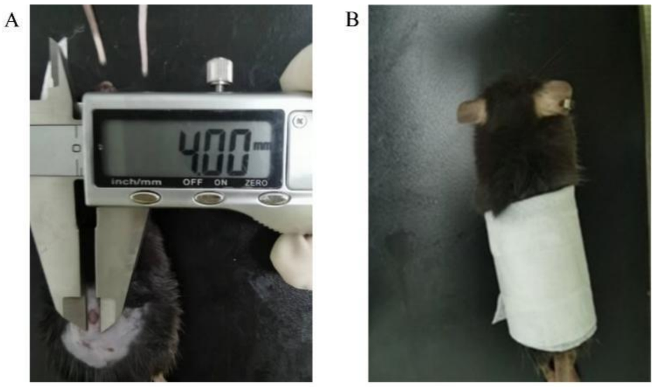

Figure S6. (A) Wound size was measured using a digital caliper, with the longest and widest distance recorded in millimeters. (B) The hydrogel was applied to the wound and fixed in place using medical tape.

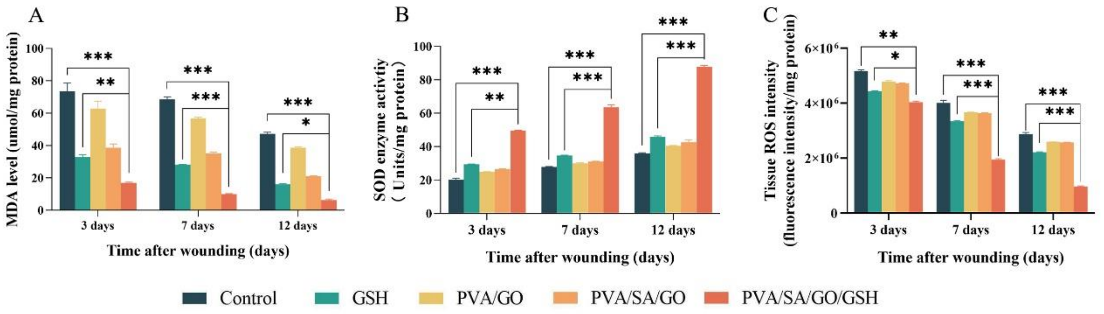

Figure S7 The quantitative analysis of oxidative stress markers in the injured tissues, including (A) MDA (n=3), (B) SOD (n=3), and (C) ROS (n=3). Data are presented as means ± standard deviation. Statistically significant differences are denoted by asterisks, with $*p \leq 0.05$, $**p \leq 0.01$, and $***p \leq 0.001$.

Table S1. Skin irritation response grading criteria[1]

skin irritation response grading	Mouse skin irritation response
**0**	no erythema and edema
**1**	slightly erythema and/or slight edema, no positive response to the touch
**2**	moderate erythema and/or edema, positive response to the touch
**3**	severe erythema and/or edema, extensive necrosis or ulceration of the skin

Table S2. Skin irritation reaction results (n=3)

Normal Control			GSH			PVA/GO			PVA/SA/GO			PVA/SA/GO/GSH		
**24h**	48h	72h	24h	48h	72h	24h	48h	72h	24h	48h	72h	24h	48h	72h
**0±0**	0±0	0±0	0±0	0±0	0±0	0±0	0±0	0±0	0±0	0±0	0±0	0±0	0±0	0±0

Reference

[1] B.S. Institution, BS EN ISO 10993-10. Biological evaluation of medical devices. Part 10. Tests for irritation and skin sensitization.
